# Genetic dissection of root traits in a rice ‘global MAGIC’ population for candidate traits to breed for reduced methane emission

**DOI:** 10.3389/fpls.2025.1616424

**Published:** 2025-07-09

**Authors:** Ripon Kumar Roy, Gopal Misra, Shaina Sharma, Bandana Pahi, Seyed Mahdi Hosseiniyan Khatibi, Kurniawan Rudi Trijatmiko, Sung-Ryul Kim, Jose E. Hernandez, Amelia Henry, Nese Sreenivasulu, Maria Genaleen Q. Diaz, Eureka Teresa M. Ocampo, Pallavi Sinha, Ajay Kohli

**Affiliations:** ^1^ International Rice Research Institute (IRRI), Los Baños, Philippines; ^2^ Bangladesh Rice Research Institute (BRRI), Gazipur, Bangladesh; ^3^ University of the Philippines Los Baños, College, Laguna, Philippines; ^4^ International Rice Research Institute, South Asia Hub, Hyderabad, Telangana, India

**Keywords:** root diameter, root porosity, genome-wide association analysis, superior haplotype, protein-protein interaction, methane emission

## Abstract

Rice cultivation is critical for global food security. The largely practiced method of rice cultivation by transplantation under flooded fields contributes significantly to methane (CH_4_) emissions, posing challenges to climate-smart agriculture. This study uses a multi-parent advanced generation inter-cross (MAGIC) population of 250 rice genotypes to understand the genetic basis of root traits that may govern CH_4_ mitigation. Phenotyping under controlled greenhouse conditions revealed significant variation in root diameter (0.122–0.481 mm) and porosity (5.344–56.793%), and strong correlations between root diameter and porosity traits (r = 0.40–0.50, p < 0.001). Association studies revealed key candidate genes including Os05g0411200 (thermosensitive chloroplast development), Os10g0177300 (chalcone synthase), and Os04g0405300 (alcohol dehydrogenase), which regulate aerenchyma formation and auxin homeostasis. Protein-protein interaction networks linked these genes to flavonoid biosynthesis (KEGG map00941) and N-glycan pathways, earlier identified as critical for root architecture. Haplotype-phenotype analysis revealed 8 superior haplotypes across 7 genes for average root porosity, base root porosity, root diameter, and tip root porosity. These findings provide the foundation for breeding high-yielding rice varieties with reduced methane emissions, addressing the challenges of food security and climate change.

## 1 Introduction

Rice is the staple food for more than 50% of the world’s population (Abeysekara and Rathnayake, 2024). Growing rice comes with the present understanding that it accounts for 12% of the global anthropogenic methane (CH_4_) emissions. Methane is a greenhouse gas (GHG) that is nearly 28 times more potent than carbon dioxide as a GHG. With the rising global population, especially in regions where rice is a staple food, meeting the growing demand for rice while reducing methane emissions from paddy fields will be an increasingly challenging task (Hertel, 2011; Sapkota et al., 2019).

Methane emission in flooded rice soils is a result of bacterial processes. These include the production and consumption of CH_4_ by methanogenic and methanotrophic bacteria, respectively, in anaerobic/aerobic microenvironments, and the oxidation of CH_4_ in aerobic microenvironments of the roots and the rhizosphere (Davamani et al., 2020; Luo et al., 2022). The rice plant plays a regulatory role in all these processes. Methane is produced by methanogenesis under reducing conditions (Kusin et al., 2025). Carbon sources in flooded rice fields provide substrates for soil methanogens, promoting a decline in soil redox potential (Eh) and creating suitable conditions for methanogen proliferation (Rago et al., 2015). Just after flooding, the soil transitions from an oxidized to a reduced state. Within a few hours to a day, electron acceptors such as O_2_, NO_3_^-^, SO_4_^-2^, Mn^4+^, and Fe^3+^ are depleted by various soil processes (Sahrawat, 2005).

Oxygen (O_2_) is utilized by aerobes and facultative anaerobes for aerobic respiration, nitrate (NO_3_^-^) by denitrifiers for denitrification, iron-reducing bacteria reduce Fe^3+^ to Fe^2+^ through iron reduction, sulfate (SO_4_^-2^) is reduced to sulfides (H_2_S, S_2_^–^, and HS^−^) by sulfate-reducing bacteria, manganese-reducing bacteria reduce Mn^4+^ to Mn^2+^, and methanogenic bacteria produce CH_4_ using CO_2_ (Ponnamperuma, 1972; Sahrawat, 2005). The correlation between pH and Eh parameters at each observation stage has a negative correlation value, indicating an inverse relationship, which enhances the methanogenic bacterial population as well as CH_4_ production (Husson, 2013). On the other hand, the reduced forms can be re-oxidized by root-released O_2_ in the rhizosphere (Yang et al., 2012). Oxygen, being the most potent oxidizing agent, ensures the availability of electron acceptors in the soil (Husson, 2013). This availability limits the food intake of methanogens, which is toxic to them (Xu et al., 2003). Consequently, the methanogenic population and its activities are inhibited, thereby hampering CH_4_ production.

Rice plants play a role in both methane production and oxidation by diffusing O_2_ to the rhizosphere (Colmer, 2003). In rice fields, methanotrophic bacteria oxidize CH_4_ as part of their energy-gaining process (Fazli et al., 2013). However, they cannot utilize CH_4_ directly in the absence of O_2_. Methane first reacts with O_2_ to produce formaldehyde, which methanotrophic bacteria then use through the ribulose monophosphate (RuMP) pathway and the serine pathway to obtain energy. This process increases the methanotrophic population, facilitating more CH_4_ oxidation. Sharp counter-gradients of oxidized and reduced species characterize the soil surface in rice fields. Where these gradients overlap, methanotrophic bacteria oxidize ≧̸90% of potentially emitted methane before it is released into the atmosphere (Reim et al., 2012).

High-yielding rice varieties with higher root porosity have greater oxidation potential as they release more oxygen into the soil (Zheng et al., 2005; Jiang et al., 2013, 2017). Atmospheric oxygen is transported from the shoot through the well-developed rice aerenchyma to the roots and finally diffuses into the rhizosphere (Mei et al., 2009; Win et al., 2011). Aerenchyma, a cortical airspace (porosity), provides a low-resistance internal pathway for the movement of O_2_ from the shoot to the roots (Armstrong, 1971, 1980). The amount of radial oxygen loss (ROL) is determined by the oxygen concentration gradient, the physical resistance to radial oxygen diffusion between the aerenchyma and the soil, and the consumption of oxygen by cells along the radial diffusion path (Ejiri et al., 2021).

The ability for radial oxygen loss (ROL) is stronger when aerenchyma is well developed, as higher root porosity enhances the oxygen diffusion to the soil (Colmer, 2003; Mei et al., 2009; Jiang et al., 2017). Root porosity, defined as the ratio of the root space volume to the mass volume of the root, is a primary factor controlling root internal O_2_ concentration and ROL (Luxmoore et al., 1970). Porosity in plant tissues results from intercellular gas-filled spaces formed during development and can be further enhanced by the formation of aerenchyma (Armstrong, 1980; Raven, 1996). Rice varieties with higher porosity tend to have higher rates of ROL (Mei et al., 2009). Root porosity is significantly associated with root diameter, with increased porosity depending on increased root diameter (Striker et al., 2007). It has been stated that root porosity depends on root diameter because a larger aerenchyma area develops in roots with greater diameter, ensuring more O_2_ secretion to the rhizosphere (Kirk, 2003; Kim et al., 2018).

From the above discussion, high-yielding rice varieties with higher radial oxygen loss capacity might be a viable option to mitigate CH_4_ emissions from rice fields. Developing such varieties requires genetic information about root diameter and porosity. Only a few genes, including *LESION SIMULATING DISEASE 1* (*LSD1*), *ENHANCED DISEASE SUSCEPTIBILITY 1* (*EDS1*), and *PHYTOALEXIN DEFICIENT 4* (*PAD4)*, have been reported to regulate lysigenous aerenchyma formation in Arabidopsis in response to hypoxia through H_2_O_2_ and ethylene signaling (Mühlenbock et al., 2007). The QTLs Qaer1.02-3, Qaer1.07, Qaer5.09, and Qaer8.06–7 have been reported for maize root aerenchyma formation under non-flooding conditions (Mano et al., 2007). However, there is a lack of studies on the genetic control (QTLs, QTNs, or genes) of rice root diameter, root porosity, and root aerenchyma.

Genome-wide association studies (GWAS) are becoming a popular method for linking genotypic variation with corresponding trait differences in several crops (Ingvarsson and Street, 2011; Xiao et al., 2017). This method facilitates the identification of major allelic variants and haplotypes in candidate genes (Sinha et al., 2020). Therefore, in the current study, we will perform GWAS to reveal the quantitative trait nucleotides (QTNs) and candidate genes responsible for higher root diameter and root porosity, aiding in marker-assisted breeding to develop low methane-emitting rice varieties.

## 2 Materials and methods

### 2.1 Plant materials

A multi-parent advanced generation inter-cross known as “global MAGIC” population of 250 inbred lines, including 234 lines and 16 parents (eight *indica* and eight *japonica*), was developed by the International Rice Research Institute (IRRI) (Bandillo et al., 2013). The elite parents used in the study were originated in the different geographical regions of the world (Colombia, China, IRRI-Philippines, India, USA, Latin America, Korea, WARDA- Ivory Coast, Uruguay) having desirable agronomic traits (high yield, good grain quality), biotic (blast and bacterial blight diseases) and abiotic (drought, salinity, submergence) stress tolerance. Experiments were carried out in the greenhouse of the International Rice Research Institute (IRRI), Los Banos, Laguna, Philippines, for 44 days (23 December 2020 to 6 February 2021 - 1st Experiment) and 40 days (26 February to 7 April 2021 - 2nd Experiment). The temperature was 26.78°C and 28.79°C, and the relative humidity was 76.88% and 71.55% in the first and second experiments, respectively, measured by HOBO Pro v2 U23-001A - Temperature/Relative Humidity Data Logger (ONSET, 470 MacArthur Blvd., Bourne, MA 02532, USA). The experiments were laid out in a Complete Randomized Design (CRD) with three replicates. Three germinated seeds were sown in a pot to ensure a single plant per pot.

### 2.2 Evaluation of the MAGIC population for the targeted traits

To investigate specific root traits that may impact methane emissions the study focused on traits such as average root porosity, base root porosity, tip root porosity, root diameter, tiller number, root number, root per tiller, shoot dry weight, root dry weight, root shoot ratio, root length, fine lateral root, thick lateral root, lateral root and nodal root on a diverse subset of 250 genotypes from the MAGIC panel (**Supplementary Table S1**). Three roots from each plant were scanned to get an 8-bit grayscale image in an Epson Perfection 7000 scanner at 600 dots per inch resolution. The images were analyzed using the WinRHIZO root system analyzer software (WinRHIZO 2004, Regent Instruments, Inc., Quebec, Canada) to generate data on average root diameter (RDia, mm). The same roots together were used to measure the root porosity traits, such as base root porosity (BP), middle root porosity (MP), and tip root porosity (TP), to minimize the error. Three cm each were taken from the base, the middle, and from the tip of the root, and the porosity was measured by following the microbalance method (Visser and Bögemann, 2003). In the case of tip porosity measurement, 3 cm was taken from 0.5 cm away from the root tip to minimize the handling error. Three cm roots were chopped into 4 equal segments to fit into a 0.5 ml microfuge tube and easily evacuate air from the root in the vacuum concentrator. We have used a 0.5 ml microfuge tube (Eppendorf) instead of a gelatin capsule for measuring the sample. Additionally root length (RL), lateral root (LR) were counted and total root dry weight (RDW) were taken.

### 2.3 Genomic data quality control

A total of 560 genotypes, including sixteen parents of the global magic population, were sequenced at an average 5x depth of coverage using the genotyping by sequencing (GBS) technique. Whole-genome sequence data of 16 parents were also present in the 3K rice project with a higher depth of coverage. Individual samples’ read data were demultiplexed into individual paired-end fastq files. Raw reads mapping and variant calling were done using a combination of BWA (Jung and Han, 2022) and GATK (McKenna et al., 2010) for every line of the population to the reference Japonica genome (version 7 of the Rice Genome Annotation Project, http://rice.plantbiology.msu.edu/).

Individual VCF files were generated for complete base calls across the entire genome for individual lines of the population, and the individual VCF files for the population were merged into a single genotype file. SNPs were filtered from this genotype file with a 30% missing rate. Single genotype files were also created for the sixteen parents, and SNPs were retained when a base pair position was present in all sixteen parents. This resulted in a set of 5 million high-quality SNPs for all sixteen parents. An SNP set with a 30% missing rate was input into the BEAGLE (Browning et al., 2018) pipeline to impute the low depth of coverage data by using parent SNP data as a reference set. The imputation process resulted in 4,40,350 SNPs across the population. Finally, 250 genotypes were selected based on genomic diversity, with 3,53,641 SNPs retained based on a 1% missing rate and a 5% minor allele frequency. The filtering method was carried out using PLINK (Purcell et al., 2007).

### 2.4 Multi-locus association mapping

Multi-locus association mapping was performed on 250 genotypes with 3,53,641 SNPs. The analysis was conducted using five different Multilocus GWAS models, including mrMLM (Wang et al., 2016), FASTmrMLM, FASTmrEMMA, ISIS EM-BLASSO, and pLARmEB (Zhang et al., 2017). The final set of significant SNPs was obtained using a threshold criterion of a LOD of 3 and above.

### 2.5 Candidate genes and functional annotation

To identify candidate genes underlying the MTAs, we considered all annotated genes located within a ±100kb length around each significant SNP, based on the estimated LD decay in the MAGIC population. Functional annotation of these genes was used as the first pass towards candidate genes, and the candidature was further refined on the basis of network analysis. The reference genome used was Oryza sativa cv. Nipponbare (IRGSP-1.0; RAP database: http://rapdb.dna.affrc.go.jp/download/irgsp1.html). The functional annotation of the candidate genes was ascertained using RAPDP (https://rapdb.dna.affrc.go.jp/), Rice Genome Annotation Project (http://rice.uga.edu/), KEGG (https://www.genome.jp/kegg/), NCBI (https://www.ncbi.nlm.nih.gov/gene), KnetMiner (https://knetminer.com/), and by reviewing the literature.

### 2.6 Haplo-pheno analysis

The total candidate genes were taken into the haplo-pheno analysis for the identification of superior haplotypes. Haplotype analysis was carried out using in-house scripts in R programming. To identify the robust donors having superior haplotypes of the key genes, best linear unbiased prediction (BLUP) analysis was done. The BLUP values were utilized for the haplo-pheno analysis. Haplo-pheno analysis was performed to associate the identified haplotypes of the selected genes with superior phenotypes. Haplotypes present in fewer than three accessions and heterozygous SNPs were removed from the analysis. The genotypes were then categorized based on haplotype groups, and superior haplotypes were identified using the phenotypic data of the individuals in each haplotype group. Then the analysis included One-way ANOVA with haplotype as a fixed factor. Subsequently, Duncan’s multiple range test (DMRT) and ANOVA were used to test the statistical significance among the means of haplotype groups using the Agricolae package in R (de Mendiburu, 2019) Groups with different letters in the graphs indicate significant differences among the groups at a p < 0.05 level of significance.

### 2.7 Protein-protein interaction network and tissue-specific expression profiling

To understand the protein interactions, String 12.0 (https://string-db.org/) (Szklarczyk et al., 2023), a database of known and predicted protein-protein interactions, was used. The input gene set was the candidate genes identified by our present study. The parameter of the PPI score was set as 0.4 (indicating medium confidence). Therefore, the PPI from String was collected for the construction of a differential protein interaction network among the candidate genes. The subset of potential genes found in the PPI network was further used for identifying Tissue-specific expression profiles using RiceXPro database, which provides high-resolution expression data across root and shoot tissues in rice (Sato et al., 2011).

## 3 Results

**Figure 1:**
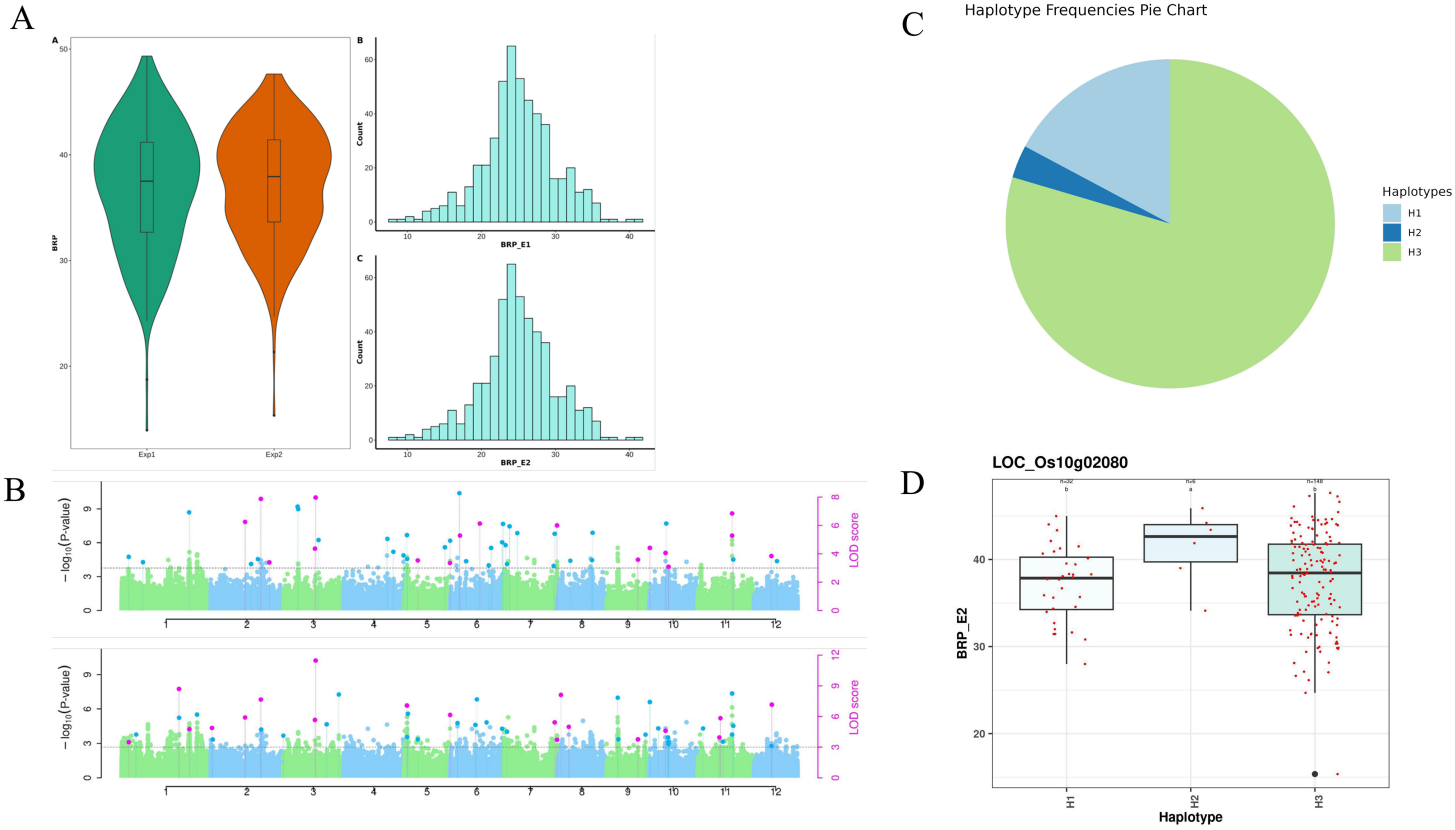
**(A)** Phenotypic diversity of base root porosity across experiments. **(B)** Manhattan plot for root diameter. **(C)** Haplotype diversity of gene LOC_Os10g02080 associated with base root porosity. **(D)** The box plot depicts the base root porosity variation among the haplotypes of gene LOC_Os10g02080.

Phenotypic values of fifteen root traits from 250 MAGIC lines were analyzed across two experiments to assess phenotypic variation. A broad range of variation was observed among the tested lines, as detailed in **Supplementary Table S1**. In the first experiment, the traits average root porosity (ARP), base root porosity (BRP), and tip root porosity (TRP) ranged from 9.457 to 47.098%, 11.41 to 53.93%, and 5.344 to 43.911%, respectively (**Figure 1A**). Root diameter (Rdia) varied from 0.122 to 0.481 mm, while root number (RN) ranged from 39 to 250. Shoot dry weight (SDW) and root dry weight (RDW) varied from 1.13 to 6.49 g and 0.18 to 1.88 g, respectively. The root-to-shoot ratio (RSRatio) ranged from 0.061 to 0.605. Root length (RL) varied from 445.265 to 2054.341 cm, and lateral root number (LR) ranged from 64.206 to 87.4.

In other experiments, ARP, BRP, and TRP ranged from 11.19 to 47.77%, 10.738 to 53.859%, and 7.168 to 56.793%, respectively. Root diameter (RDia) varied from 0.122 to 0.449 mm, while root number (RN) ranged from 79 to 332. Shoot dry weight (SDW) and root dry weight (RDW) varied from 2.69 to 8.48 g and 0.37 to 3.11 g, respectively. The root-to-shoot ratio (RSRatio) ranged from 0.11 to 0.58. Root length (RL) varied from 343.421 to 1496.119 cm, and lateral root number (LR) ranged from 64.448 to 86.745.

The standard deviation ranges between the first and second experiments demonstrated minimal variation for traits such as TN (1.893-1.635), RPT (6.320-7.025), SDW (0.840-0.919), RDW (0.273-0.355), RS Ratio (0.071-0.055), RDia (0.051-0.052), BRP (6.656-6.788), TRP (6.236-6.141), ARP (5.460-5.284), FLR (6.092-5.415), TLR (4.651-4.275), LR (3.471-3.421), and NR (3.471-3.421). In contrast, traits such as RN (32.303-44.721) and RL (168.080-158.594) exhibited greater standard deviation ranges, indicating a higher degree of diversity within the Global MAGIC population (**Supplementary Table S1**). The frequency distributions of phenotypes revealed continuous distributions, characteristic of quantitative traits, in both experiments. These results demonstrate significant phenotypic variation in root traits and methane emissions, providing a foundation for further genetic and environmental impact studies.

### 3.1 Correlation among the traits

Correlation trends among the studied traits were consistent across both experiments (**Supplementary Figures S1**, **S2**). RN exhibited strong correlations with RPT, RS ratio, TN, and RDW. Root porosity traits (BRP, TRP, and ARP) showed significant correlations with RDia. The correlation values of RL and FLR with NR were -0.38 and -0.61, respectively, indicating negative correlations. The correlation values between BRP and RDia, TRP and RDia, and ARP and RDia were approximately 0.45, 0.40, and 0.50, respectively. Additionally, BRP, TRP, and ARP were positively correlated, with ARP being the average of BRP and TRP. The correlation values between TRP and BRP, TRP and ARP, and BRP and ARP were around 0.45, 0.85, and 0.90, respectively. These results suggest that candidate genes associated with any of the studied traits could effectively enhance root porosity.

### 3.2 Unveiling significant MTAs through multi-locus GWAS

GWAS analysis was done using 3,49,594 SNPs on 250 genotypes to unveil meaningful MTAs. Employing five multilocus GWAS methods mrMLM, FASTmrMLM, FASTmrEMMA, ISIS EM-BLASSO, and pLARmEB, significant associations were identified by considering a LOD value ≥3 along with the phenotypic variance (PVE) as a threshold for significance. In total, GWAS successfully identified 193 and 195 significant QTNs in the 1st and 2nd experiments, respectively, associated with four root traits (RDia, BRP, TRP, and ARP) across the 12 chromosomes in all four multi-locus GWAS methods.

For Average root porosity (ARP), a total of 48 and 41 significant MTAs were identified from the GWAS analysis in both experiments, respectively. These MTAs were distributed across all chromosomes in both experiments (**Supplementary Table S2**). Among these significant MTAs, 30 and 25 were found within the genic regions in both experiments, respectively (**Supplementary Table S3**). The phenotypic variance explained (PVE) by the MTAs ranged from 0.34% to 12.59% in 1^st^ experiment and 0.2% to 12.55% in 2^nd^ experiment. Furthermore, we examined the consistency of MTAs between the two experiments and identified 12 common MTAs. The soundness of the model is evident from the well-fitted Manhattan and Q-Q plots (**Supplementary Figure S3**).

For Base root porosity, a total of 100 significant MTAs were identified from the GWAS analysis in both experiments. These MTAs were distributed across all chromosomes in both experiments except chromosome 4 in 2^nd^ experiment (**Figure 1B**, **Supplementary Table S4**). Among these significant MTAs, 29 were found within the genic regions in both experiments (**Supplementary Table S5**). The phenotypic variance explained (PVE) by the MTAs ranged from 0.34% to 10.71% in 1^st^ experiment and 0.23% to 9.67% in 2^nd^ experiment. Furthermore, we examined the consistency of MTAs between the two experiments and identified 20 common MTAs. The soundness of the model is evident from the well-fitted Manhattan and Q-Q plots (**Supplementary Figure S4**).

For root diameter, a total of 45 and 60 significant MTAs were identified from the analysis in the 1st and 2nd experiments, respectively (**Figure 1B**). These MTAs were distributed across all chromosomes in both experiments except chromosome 11 in 1st experiment (**Supplementary Table S6**). Among these significant MTAs, 20 and 30 were found within the genic regions in both experiments respectively (**Supplementary Table S7**). The phenotypic variance explained (PVE) by the MTAs ranged from 0.74% to 10.2% in 1^st^ experiment and 0.00% to 9.57% in 2^nd^ experiment. Furthermore, we examined the consistency of MTAs between the two experiments and identified 11 common MTAs. The soundness of the model is evident from the well-fitted Manhattan and Q-Q plots (**Supplementary Figure S5**).

For tip root porosity, a total of 50 and 44 significant MTAs were identified from the analysis in both experiments, respectively. These MTAs were distributed across all chromosomes except chromosome 5 in both experiments (**Supplementary Table S8**). Among these significant MTAs, 29 and 27 were found within the genic regions in both experiments respectively (**Supplementary Table S9**). The phenotypic variance explained (PVE) by the MTAs ranged from 0.22% to 10.65% in 1^st^ experiment and 1.01% to 16.24% in 2^nd^ experiment. Furthermore, we examined the consistency of MTAs between the two experiments and identified 13 common MTAs. The soundness of the model is evident from the well-fitted Manhattan and Q-Q plots (**Supplementary Figure S6**).

For Tiller number (TN), a total of 44 and 48 significant MTAs were identified from the analysis in the first and second experiments respectively, spanning all chromosomes (**Supplementary Table S10**). Among these significant MTAs, 25 and 30 were located within genic regions in both experiments respectively (**Supplementary Table S11**). The phenotypic variance explained (PVE) by the MTAs ranged from 0.9% to 9.1% in the first experiment and 0.67% to 9.4% in the second experiment. The robustness of the model is evidenced by the well-fitted Manhattan and Q-Q plots (**Supplementary Figure S7**). Similarly, for root number (RN), a total of 55 and 52 significant MTAs were identified from the analysis in the first and second experiments respectively, spread across all chromosomes (**Supplementary Table S12**). Among these significant MTAs, 24 were present within genic regions in both the experiments (**Supplementary Table S13**). The phenotypic variance explained (PVE) by the MTAs ranged from 0.48% to 11.6% in the first experiment and 0.38% to 8.57% in the second experiment. The reliability of the model is apparent from the well-fitted Manhattan and Q-Q plots (**Supplementary Figure S8**).

For root per tiller (RPT), a total of 39 and 56 significant MTAs identified from the analysis in 1^st^ and 2^nd^ experiments respectively. These MTAs were distributed across all chromosomes in both experiments except 3 and 9 chromosomes in 1^st^ experiment (**Supplementary Table S14**). Among these significant MTAs, 26 and 24 were found within the genic regions in both experiments respectively (**Supplementary Table S15**). The phenotypic variance explained (PVE) by the MTAs ranged from 0.47% to 10.63% in 1^st^ experiment and 0.34% to 8.25% in 2^nd^ experiment. The soundness of the model is evident from the well-fitted Manhattan and Q-Q plots (**Supplementary Figure S9**). For shoot dry weight (SDW), a total of 38 and 61 significant MTAs identified from the analysis in 1^st^ and 2^nd^ experiments respectively. These MTAs were distributed across all chromosomes in both experiments except chromosome 1 in 1st experiment (**Supplementary Table S16**). Among these significant MTAs, 20 and 37 were found within the genic regions in both experiments respectively (**Supplementary Table S17**). The phenotypic variance explained (PVE) by the MTAs ranged from 0.26% to 12.01% in 1st experiment and 3.8% to 11.55% in 2nd experiment. The soundness of the model is evident from the well-fitted Manhattan and Q-Q plots (**Supplementary Figure S10**).

For root dry weight (RDW), a total of 41 and 49 significant MTAs identified from the analysis in 1st and 2nd experiments respectively. Both experiments distributed These MTAs across all chromosomes (**Supplementary Table S18**). Among these significant MTAs, 21 and 32 were found within the genic regions in both experiments respectively (**Supplementary Table S19**). The phenotypic variance explained (PVE) by the MTAs ranged from 1.33% to 9.23% in 1^st^ experiment and 0.62% to 10.2% in 2^nd^ experiment. Furthermore, we examined the consistency of MTAs between the two experiments and identified 2 common MTAs. The soundness of the model is evident from the well-fitted Manhattan and Q-Q plots (**Supplementary Figure S11**). For root shoot ratio (RS Ratio), a total of 51 and 58 significant MTAs identified from the analysis in 1^st^ and 2^nd^ experiments respectively. Both experiments distributed These MTAs across all chromosomes (**Supplementary Table S20**). Among these significant MTAs, 32 and 31 were found within the genic regions in both experiments respectively (**Supplementary Table S21**). The phenotypic variance explained (PVE) by the MTAs ranged from 0% to 10.97% in 1^st^ experiment and 0.04% to 10.13% in 2^nd^ experiment. Furthermore, we examined the consistency of MTAs between the two experiments and identified 1 common MTAs. The soundness of the model is evident from the well-fitted Manhattan and Q-Q plots (**Supplementary Figure S12**).

For root length (RL), a total of 65 and 60 significant MTAs identified from the analysis in 1^st^ and 2^nd^ experiments respectively. These MTAs were distributed across all chromosomes in both experiments (**Supplementary Table S22**). Among these significant MTAs, 30 and 31 were found within the genic regions in both experiments respectively (**Supplementary Table S23**). The phenotypic variance explained (PVE) by the MTAs ranged from 0.5% to 9.2% in 1^st^ experiment and 0.91% to 10.4% in 2^nd^ experiment. Furthermore, we examined the consistency of MTAs between the two experiments and identified 9 common MTAs. The soundness of the model is evident from the well-fitted Manhattan and Q-Q plots (**Supplementary Figure S13**). For fine lateral root (FLR), a total of 39 and 51 significant MTAs identified from the analysis in 1st and 2nd experiments respectively. These MTAs were distributed across all chromosomes in both experiments (**Supplementary Table S24**). Among these significant MTAs, 19 and 23 were found within the genic regions in both experiments respectively (**Supplementary Table S25**). The phenotypic variance explained (PVE) by the MTAs ranged from 0.9% to 12.08% in 1^st^ experiment and 0.6% to 9.02% in 2^nd^ experiment. Furthermore, we examined the consistency of MTAs between the two experiments and identified 8 common MTAs. The soundness of the model is evident from the well-fitted Manhattan and Q-Q plots (**Supplementary Figure S14**).

For thick lateral root (TLR), a total of 49 and 35 significant MTAs identified from the analysis in 1^st^ and 2^nd^ experiments respectively. These MTAs were distributed across all chromosomes in both experiments except chromosome 9 in 2nd experiment (**Supplementary Table S26**). Among these significant MTAs, 31 and 20 were found within the genic regions in both experiments respectively (**Supplementary Table S27**). The phenotypic variance explained (PVE) by the MTAs ranged from 0.96% to 9.8% in 1^st^ experiment and 0.2% to 10.4% in 2^nd^ experiment. Furthermore, we examined the consistency of MTAs between the two experiments and identified 8 common MTAs. The soundness of the model is evident from the well-fitted Manhattan and Q-Q plots (**Supplementary Figure S15**).

For lateral root (LR), a total of 53 and 50 significant MTAs identified from the analysis in 1^st^ and 2^nd^ experiments respectively. These MTAs were distributed across all chromosomes in both experiments (**Supplementary Table S28**). Among these significant MTAs, 31 and 32 were found within the genic regions in both experiments respectively (**Supplementary Table S29**). The phenotypic variance explained (PVE) by the MTAs ranged from 0.75% to 9.6% in 1^st^ experiment and 0.73% to 12.83% in 2nd experiment. Furthermore, we examined the consistency of MTAs between the two experiments and identified 6 common MTAs. The soundness of the model is evident from the well-fitted Manhattan and Q-Q plots (**Supplementary Figure S16**).

For Nodal root (NR), a total of 48 and 55 significant MTAs identified from the analysis in 1st and 2nd experiments respectively. These MTAs were distributed across all chromosomes in both experiments except in chromosome 6 in 1st experiment (**Supplementary Table S30**). Among these significant MTAs, 30 and 33 were found within the genic regions in both experiments respectively (**Supplementary Table S31**). The phenotypic variance explained (PVE) by the MTAs ranged from 1.32% to 9.8% in 1st experiment and 1.2% to 9.9% in 2nd experiment. Furthermore, we examined the consistency of MTAs between the two experiments and identified 6 common MTAs. The soundness of the model is evident from the well-fitted Manhattan and Q-Q plots (**Supplementary Figure S17**).

### 3.3 Prediction and annotation of potential candidate genes

The analysis of 15 targeted traits across two experiments, examining the number of MTAs, genic MTAs, and expected PVE ranges is presented in **Table 1**. ARP yielded 197 candidate genes from 48 significant MTAs in 1^st^ experiment and 148 candidate genes from 41 significant MTAs in 2^nd^ experiment. For BRP, 257 candidate genes emerged from 50 significant MTAs in the initial experiment, with a similar count of 255 candidate genes from 50 significant MTAs in the subsequent experiment. In the case of RDia, the first experiment identified 189 candidate genes from 45 significant MTAs, while the second experiment revealed 323 candidate genes from 60 significant MTAs. TRP showcased 203 candidate genes from 50 significant MTAs in the first experiment, contrasting with 155 candidate genes from 44 significant MTAs in the second experiment. In the first experiment, TN yielded 233 candidate genes from 44 significant MTAs, while the second experiment identified 189 candidate genes from 48 significant MTAs.

**Table 1:** List of identified genic MTAs, candidate genes and PVE % for all traits.

Trait	Experiment	No of MTAs	No of Candidate genes	PVE (%)
Average Root Porosity	Exp1	48	30	0.34 - 12.59
Average Root Porosity	Exp2	41	25	0.20 - 12.55
Base Root Porosity	Exp1	50	29	0.34 - 10.71
Base Root Porosity	Exp2	50	29	0.23 - 09.67
Root Diameter	Exp1	45	20	0.74 - 10.20
Root Diameter	Exp2	60	30	0.00 - 09.57
Tip Root Porosity	Exp1	50	29	0.22 - 10.65
Tip Root Porosity	Exp2	44	27	1.01 - 16.24
Tiller Number	Exp1	44	25	0.90 - 09.10
Tiller Number	Exp2	48	30	0.67 - 09.40
Root Number	Exp1	55	24	0.48 - 11.60
Root Number	Exp2	52	24	0.38 - 08.57
Root Per Tiller	Exp1	39	26	0.47 - 10.63
Root Per Tiller	Exp2	56	54	0.34 - 08.25
Shoot Dry Weight	Exp1	38	20	0.26 - 12.01
Shoot Dry Weight	Exp2	61	37	3.82 - 11.55
Root Dry Weight	Exp1	41	21	1.33 - 09.23
Root Dry Weight	Exp2	49	32	0.62 - 10.20
Root Shoot Ratio	Exp1	51	32	0.00 - 10.97
Root Shoot Ratio	Exp2	58	31	0.04 - 10.13
Root Length	Exp1	65	30	0.50 - 09.20
Root Length	Exp2	60	31	0.91 - 10.40
Fine Lateral Root	Exp1	39	19	0.90 - 12.08
Fine Lateral Root	Exp2	51	23	0.60 - 09.02
Thick Lateral	Exp1	49	31	0.96 - 09.80
Thick Lateral	Exp2	35	20	0.20 - 10.40
Lateral Root	Exp1	53	31	0.75 - 09.60
Lateral Root	Exp2	50	32	0.73 - 12.86
Nodal Root	Exp1	48	30	1.32 - 09.80
Nodal Root	Exp2	55	33	1.20 - 09.90

Similarly, RN reveled 256 candidate genes from 55 significant MTAs in the first experiment and 236 candidate genes from 52 significant MTAs in the second experiment. RPT resulted in 193 candidate genes from 39 significant MTAs initially and 275 candidate genes from 56 significant MTAs subsequently. In the case of SDW, 172 candidate genes from 38 significant MTAs and 344 candidate genes from 61 significant MTAs were identified from the first and second experiments respectively. The RDW trait showed 181 candidate genes from 41 significant MTAs in the first experiment and 276 candidate genes from 49 significant MTAs in the second experiment. Further, RS ratio unveiled 268 candidate genes from 51 significant MTAs in the first experiment and 271 candidate genes from 58 significant MTAs in the second experiment. Lastly, RL exhibited 239 candidate genes from 65 significant MTAs in the first experiment and 258 candidate genes from 60 significant MTAs in the second experiment.

For FLR, the first experiment identified 187 candidate genes from 39 significant MTAs, while the second experiment reveled 253 candidate genes from 51 significant MTAs. In the case of TLR, the initial experiment uncovered 297 candidate genes from 49 significant MTAs, whereas the subsequent experiment yielded 165 candidate genes from 35 significant MTAs. LR analysis showcased 233 candidate genes from 53 significant MTAs and 205 candidate genes from 50 significant MTAs from the first and second experiments, respectively. Similarly, a thorough investigation revealed 203 candidate genes from 48 significant MTAs and 273 candidate genes from 55 significant MTAs associated with NR in the first and second experiments respectively. All candidate genes were annotated using the SnpEff software.

### 3.4 Identification of superior haplotypes

To further explore the functional implications of these genetic loci, we investigated haplotype-level variation and gene expression dynamics among the identified candidate genes. A total of 219 candidate genes, 55,58,50, and 56 genes showed MTAs for the traits ARP, BRP, RDia, and TRP, respectively, in both experiments (**Table 1**). Among these, 7 of these genes exhibited superior haplotypes while the remaining genes showed no variation across the 250 MAGIC lines. The distribution of haplotypes associated with target traits reveals a notable variation across different loci. A total of 8 superior haplotypes were identified for seven target genes associated with these traits. The distribution of lines for superior haplotype across the identified genes ranges from 38 to 76 in ARP, 6 to 76 in BRP,18 to 143 in RDia and 1 to 76 in TRP. Meanwhile, the range of frequency distribution of the targeted traits was as low as 0.03 in LOC_Os10g02080-H2 for BRP (**Figures 1C, D**), while as high as 0.77 in LOC_Os01g29250-H1 for RDia. Frequency distribution for the observed traits varied, with ARP ranging from 0.29 (LOC_Os01g39740-H5) to 0.44 (LOC_Os07g02630-H1), BRP from 0.44 (LOC_Os07g02630-H1) to 0.03 (LOC_Os10g02080-H2), RDia from 0.11 (LOC_Os11g40860-H1) to 0.77 (LOC_Os01g29250-H1) and for TRP 0.44 (LOC_Os07g02630-H1) (**Supplementary Table S32**). The diversity analysis of haplotypes across identified MTA can provide valuable insight into the genetic variation of the population.

### 3.5 Network analysis of candidate genes associated with rice roots

**Figure 2:**
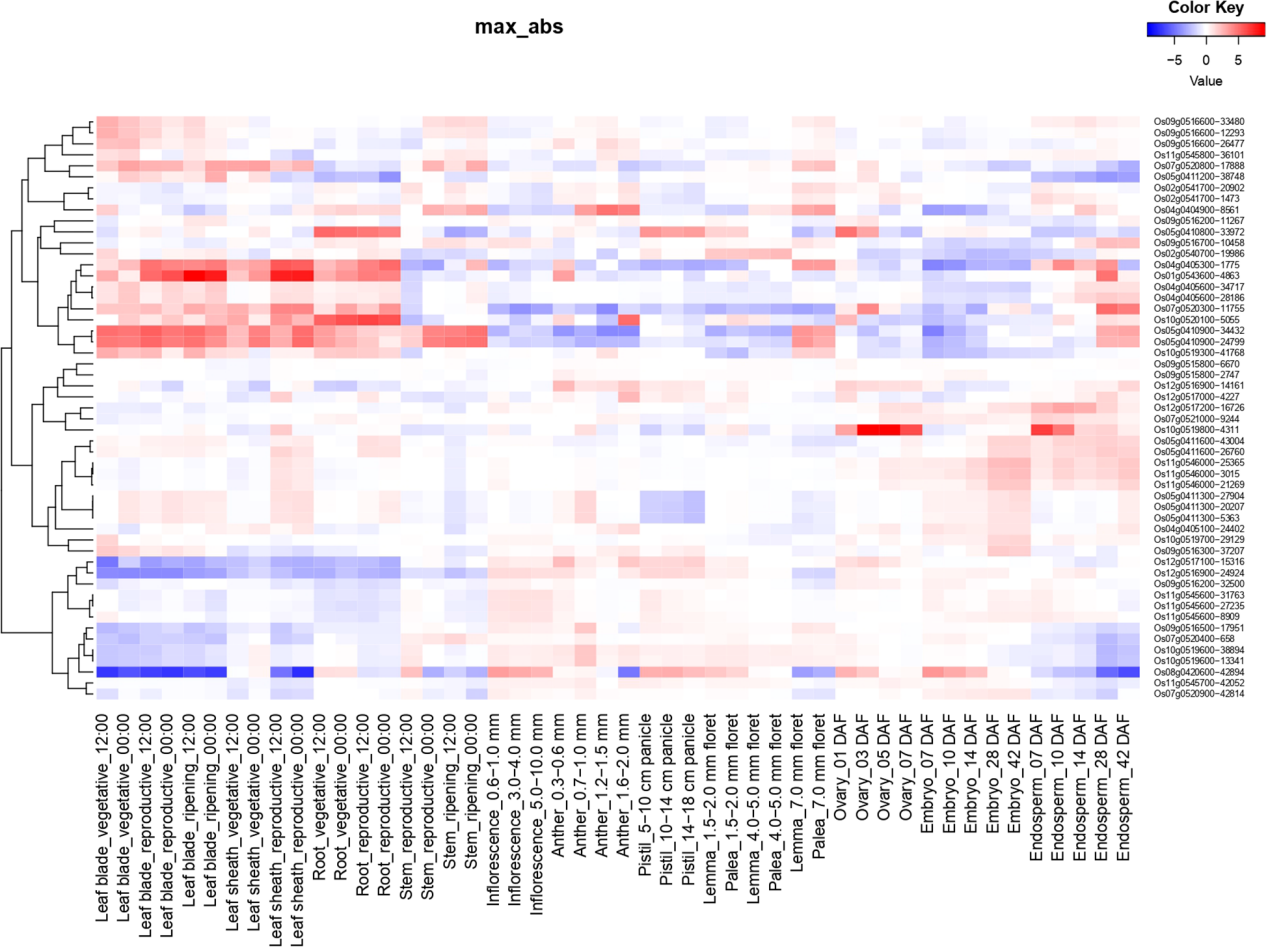
Tissue-specific expression profiles of candidate genes associated with root traits using RiceXPro. Expression patterns of 16 candidate genes identified through GWAS were examined across various rice tissues. The heatmap illustrates gene expression intensity based on median-centered values generated from RiceXPro, with a focus on root tissue.

**Figure 3:**
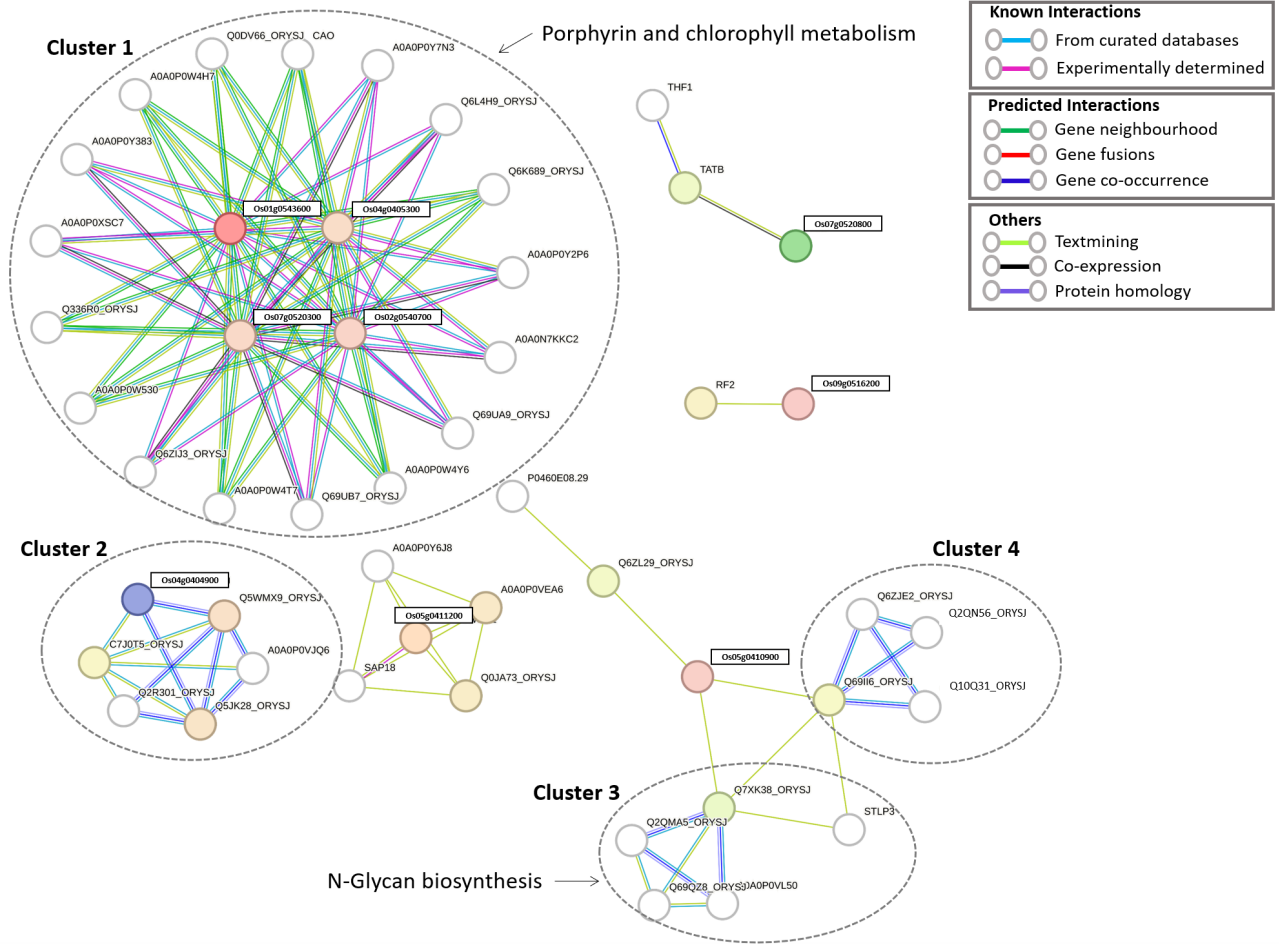
Protein-protein interaction (PPI) network of 16 candidate genes involved in root development. The network consists of three primary clusters and three auxiliary subnetworks.

Expression of identified candidate genes using RiceXPro (https://ricexpro.dna.affrc.go.jp/) (Sato et al., 2013) in various tissues (**Figure 2**) showed 16 differentially expressed genes in root tissues. This gene set consists of many genes that are involved in plant growth and development, such as U-box containing protein, basic Leucine Zipper Domain (bZIP) containing transcription factor, F-box proteins, and cytochrome P450 genes (Abd-Hamid et al., 2020; Mao et al., 2022; Han et al., 2023), and the details of the identified genes are given in **Supplementary Table S33**. We have performed a protein-protein interaction network analysis of these genes, and **Figure 3** shows the interaction network as determined by the STRING database (Szklarczyk et al., 2023) (version 11.0; https://string-db.org/). We observed 3 main clusters with three auxiliary networks that consist of 9 genes from our queries. The main cluster has 4 of the query genes and though most of the genes in this cluster are involved in the porphyrin and chlorophyll metabolism some genes belong to the Chalcone/stilbene synthases family genes (A0A0P0XSC7: Os10g0177300, Q6L4H9_ORYSJ: Os05g0212500, and A0A0N7KKC2: Os05g0212600) that are known for their involvement in flavonoid biosynthesis and in the regulation of auxin transport and in the root gravitropism (Hu et al., 2021). Two other clusters (2 & 4) mainly consist of putative genes, and they are found to be interacting with one of the peptide transferase proteins (Os05g0410900) we identified. The second cluster has genes that are all involved in the N-glycan biosynthesis pathways, which have an effect on the cellulose content (Strasser, 2014). The genes in this cluster are interacting with one of the candidate genes, Os05g0411200 (*OsTCD5*). It is a thermosensitive chloroplast development gene. The details of the genes identified in this network and the interaction score for this network are given in **Supplementary Tables S34**, **S35**, respectively.

**Figure 4:**
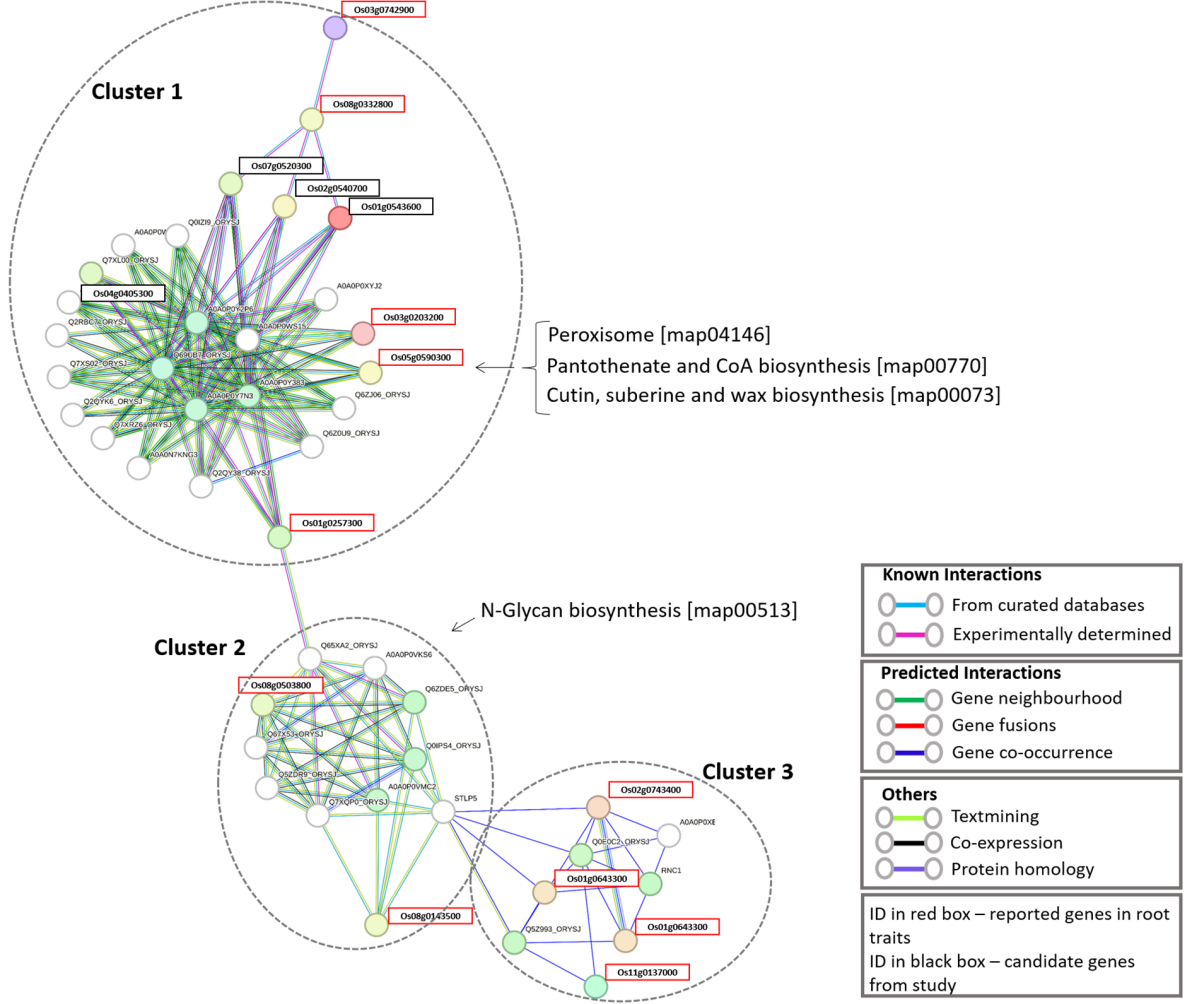
Combined protein-protein interaction network of candidate genes and reported root development genes.

**Figure 5:**
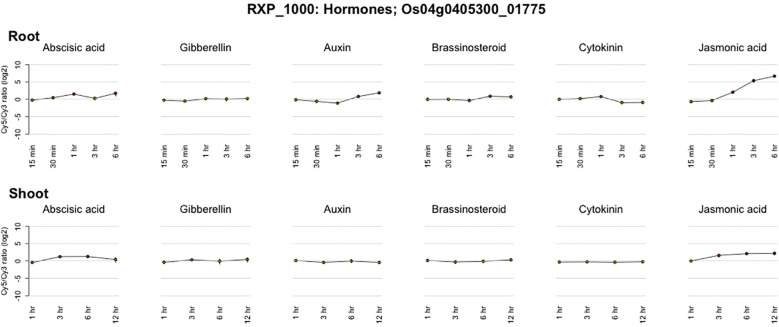
Temporal expression pattern of *Os04g0405300* in response to hormonal cues (Sato et al., 2013).

To determine how the identified candidate genes interact with reported root trait-related genes, we retrieved 26 proteins already known to be involved in root development and functionality from the literature (Wimalagunasekara et al., 2023) and the oryzabase database (https://shigen.nig.ac.jp/rice/oryzabase/). The details of these reported genes are given in **Supplementary Table S36**. The combined input of the 26 reported genes and the 16 candidate genes, resulted in a fully connected network with 3 distinct clusters (**Figure 4**). Four of the candidate genes were found to be interacting with 11 of the reported genes. All four of the candidate genes are present in the first cluster, which contains genes involved in three major pathways (Peroxisome, Pantothenate and CoA biosynthesis, and Cutin, suberine and wax biosynthesis). The second cluster has two reported genes which are involved in the N-glycan biosynthesis pathway. The third cluster contains 4 reported genes. Based on the genes present in the closed cluster, we can interpret that the 4 candidate genes might have a role in root branching and/or the number of adventitious and lateral roots. The details of the genes identified in this network and the interaction score for this network are given in **Supplementary Tables S37**, **S38**, respectively. Interestingly, one gene (Os04g0405300) among the 4 candidate genes showed a significant difference in auxin and Jasmonic acid production with respect to the different periods of time (**Figure 5**). This gene regulates alcohol dehydrogenase/reductase, which is crucial for maintaining homeostasis of active auxin levels within the plants.

## 4 Discussion

O_2_ plays a regulatory role in CH_4_ production, oxidation, and emission from flooded rice fields (Colmer, 2003; Yang et al., 2012; Fazli et al., 2013). Atmospheric O2 reaches the rhizospheric soil through the well-developed rice aerenchyma system (Armstrong, 1971, 1980). In most cases, O2 diffusion into the soil depends on the root’s radial oxygen loss capacity and porosity (Luxmoore et al., 1970). Root porosity generally depends on aerenchyma formation and root diameter (Armstrong, 1980; Jeffree and Fry, 1986). These factors are crucial because the root acts as a conduit, holding and transporting various gases (O_2_, CH_4_, CO_2_, N_2_O) between the atmosphere and the soil. Recent studies continue to emphasize the importance of these traits in managing greenhouse gas emissions from rice paddies (Mei et al., 2009; Jiang et al., 2017; Zheng et al., 2018).

Our study enabled the identification of essential QTNs and genes located within these QTNs. We evaluated the Global Multi-parent Advanced Generation Inter-Cross (MAGIC) population, focusing on four traits related to root porosity and diameter: RDia, BP, TP, and ARP. Notably, significant variations were observed among these traits and genotypes. This diversity could be attributed to the use of diverse genotypes derived from multiple elite parents across the globe. The Global MAGIC population, generated from eight *indica* and eight *japonica* patents, integrates multiple traits from both gene pools, which have adapted to diverse ecotypes worldwide. Recombination among the 16 genotypes resulted in novel genetic variation. Key advantages of the global MAGIC population include ample genetic diversity, a higher allelic balance frequency compared to biparental populations, and minimal impact on population structure (Valdar et al., 2006; Mackay and Powell, 2007; Kover et al., 2009). Additionally, it enhances mapping accuracy through historical and synthetic recombination (Meng et al., 2016).

The evaluation of root traits across two experiments using the Global MAGIC population revealed a consistent pattern of phenotypic variation, reinforcing the reliability of the observed trait distribution. However, the minor variation detected, particularly in root number and root length, reflects the plastic nature of root architecture, which is likely due to environmental influence. In our study, we observed significant correlations among the traits related to root porosity: basal root porosity, total root porosity, and apical root porosity. These porosity traits were strongly associated with root diameter (RDia). Previous research by Striker et al. (2007) reported that increased root porosity is directly dependent on larger root diameter. Additionally, we found that BRP, TRP, and ARP were closely linked. Root porosity is intricately tied to the formation of root aerenchyma. In rice, lysigenous aerenchyma forms in the cortex through cell death and subsequent lysis (Armstrong, 1980; Jackson et al., 1985). The process begins in mid-cortex cells and spreads radially to surrounding cortical cells (Kawai et al., 1998). Aerenchyma formation initiates at the apical parts of rice roots and gradually extends to the basal regions (Ranathunge et al., 2003). Ultimately, fully developed aerenchyma is observed at the basal part of the root (Armstrong and Armstrong, 1994; Kozela and Regan, 2003; Ranathunge et al., 2003). Despite the remarkable difference in BRP compared to TRP, these traits remain closely associated. These factors had a direct or indirect positive effect on the amount of oxygen diffused into the rhizosphere, promoting methane oxidation.

We utilized 250 global MAGIC population lines, each characterized by 3,53,641 Single SNPs. Our goal was to identify QTNs and candidate genes associated with root diameter and root porosity. To achieve this, multi-locus GWAS models outperform single-locus models statistically, with a lower False Positive Rate (FPR) (Segura et al., 2012; Wang et al., 2016; Zhang et al., 2019). These models closely resemble true genetic architectures in plants and animals. By incorporating polygenic effects and accounting for population structure, multi-locus models mitigate bias in effect estimation (Zhang et al., 2005, 2010; Yu et al., 2006). The five multi-locus GWAS methods in this study is an integration of FASTmrEMMA (Wen et al., 2018), ISIS EM-BLASSO (Tamba et al., 2017) mrMLM (Wang et al., 2016), pLARmEB (Zhang et al., 2017) and pKWmEB (Ren et al., 2018).

The present study has identified a substantial number of MTAs across key root traits in rice, with candidate genes ranging from 19 to 54 for each trait (**Table 1**). PVE values were generally low to moderate (0.2% to 16%), indicating that these traits are influenced by many small-effect loci rather than a few significant genes. We filtered down from 274 candidate genes for Rdia, ARP, BRP and TRP to 16 candidate genes based on the expression of the identified genes in root tissues. The protein-protein interaction of these genes revealed three primary clusters (**Figure 3**). The primary cluster includes key genes regulating chlorophyll metabolism, flavonoid biosynthesis, and affecting auxin transport. While another cluster, 2 and 4, has putative genes interacting with peptide transferase. Combining GWAS with protein-protein analysis enhances the validation of identified MTAs and deepens the understanding of the molecular framework regulating traits of interest.

## 5 Conclusion

This study provides critical insights into the genetic architecture of rice root traits that can mitigate methane emissions in paddy ecosystems. By utilizing a genetically diverse Global MAGIC population and GWAS, we identified several MTAs and candidate genes associated with root diameter and porosity, two traits directly influencing root-mediated oxygen transport and CH_4_ oxidation. Our haplotype-phenotype analysis identified eight superior haplotypes across seven key genes, with wide variation in frequency, confirming substantial allelic diversity within the population. Furthermore, network analysis of 16 root-expressed candidate genes revealed their functional integration with known root developmental pathways, including auxin regulation, flavonoid biosynthesis, and cell wall modification, reinforcing their biological significance. Together, these results establish a comprehensive framework for selecting and developing rice varieties with improved root traits and lower greenhouse gas emissions, paving the way for sustainable and climate-smart rice cultivation.

## Data Availability

The datasets presented in this study can be found in online repositories. The names of the repository/repositories and accession number(s) can be found in the article/**Supplementary Material**.

## Supplementary material

Supplementary Figure 1 Phenotypic variation and correlation among traits in experiment 1. The correlation plot compares Average Root Porosity (ARP), Base Root Porosity (BRP), Root Diameter (RDia), Tip Root Porosity (TRP), Tiller Number (TN), Root Number (RN), Root Per Tiller (RPT), Shoot Dry Weight (SDW), Root Dry Weight (RDW), Ratio Between Root Shoot (RS ratio), Root Length (RL), Fine Lateral Root (FLR), Thick Lateral Root (TLR), Lateral Root (LR) and Nodal Root (NR). Bar charts show the frequency distributions of each trait on the diagonal. Pearson correlation values are above the diagonal with their corresponding P-values, and scatter plots are below the diagonal. Data correspond to mean values of 250 MAGIC population in two different experiments **(a, b)**. Symbols indicate the significance levels: * **P < 0.01 and ***P < 0.005.

Supplementary Figure 2 Phenotypic variation and correlation among traits in experiment 2. The correlation plot compares Average Root Porosity (ARP), Base Root Porosity (BRP), Root Diameter (RDia), Tip Root Porosity (TRP), Tiller Number (TN), Root Number (RN), Root Per Tiller (RPT), Shoot Dry Weight (SDW), Root Dry Weight (RDW), Ratio Between Root Shoot (RS ratio), Root Length (RL), Fine Lateral Root (FLR), Thick Lateral Root (TLR), Lateral Root (LR) and Nodal Root (NR). Bar charts show the frequency distributions of each trait on the diagonal. Pearson correlation values are above the diagonal with their corresponding P-values, and scatter plots are below the diagonal. Data correspond to mean values of 250 MAGIC population in two different experiments **(a, b)**. Symbols indicate the significance levels: * **P < 0.01 and ***P < 0.005.

Supplementary Figure 3
**(A)** Phenotypic diversity of population structure and average root porosity of 250 accessions. **(B)** Manhattan plot for root length **(C)** Q-Q plot for average root porosity.

Supplementary Figure 4
**(A)** Phenotypic diversity of population structure and base root porosity of 250 accessions. **(B)** Manhattan plot for base root porosity **(C)** Q-Q plot for base root porosity.

Supplementary Figure 5
**(A)** Phenotypic diversity of population structure and root diameter of 250 accessions. **(B)** Manhattan plot for root diameter **(C)** Q-Q plot for root diameter.

Supplementary Figure 6
**(A)** Phenotypic diversity of population structure and tip root porosity of 250 accessions. **(B)** Manhattan plot for tip root porosity **(C)** Q-Q plot for tip root porosity.

Supplementary Figure 7
**(A)** Phenotypic diversity of population structure and tiller number of 250 accessions. **(B)** Manhattan plot for tiller number **(C)** Q-Q plot for tiller number.

Supplementary Figure 8
**(A)** Phenotypic diversity of population structure and root number of 250 accessions. **(B)** Manhattan plot for root number **(C)** Q-Q plot for root number.

Supplementary Figure 9
**(A)** Phenotypic diversity of population structure and root per tiller of 250 accessions. **(B)** Manhattan plot for root per tiller **(C)** Q-Q plot for root per tiller.

Supplementary Figure 10
**(A)** Phenotypic diversity of population structure and shoot dry weight of 250 accessions. **(B)** Manhattan plot for shoot dry weight **(C)** Q-Q plot for shoot dry weight.

Supplementary Figure 11
**(A)** Phenotypic diversity of population structure and root dry weight of 250 accessions. **(B)** Manhattan plot for root dry weight **(C)** Q-Q plot for root dry weight.

Supplementary Figure 12
**(A)** Phenotypic diversity of population structure and root shoot ratio of 250 accessions. **(B)** Manhattan plot for root shoot ratio **(C)** Q-Q plot for root shoot ratio.

Supplementary Figure 13
**(A)** Phenotypic diversity of population structure and root length of 250 accessions. **(B)** Manhattan plot for root length **(C)** Q-Q plot for root length.

Supplementary Figure 14
**(A)** Phenotypic diversity of population structure and fine lateral root of 250 accessions. **(B)** Manhattan plot for fine lateral root **(C)** Q-Q plot for fine lateral root.

Supplementary Figure 15
**(A)** Phenotypic diversity of population structure and thick lateral root of 250 accessions. **(B)** Manhattan plot for thick lateral root **(C)** Q-Q plot for thick lateral root.

Supplementary Figure 16
**(A)** Phenotypic diversity of population structure and lateral root of 250 accessions. **(B)** Manhattan plot for lateral root **(C)** Q-Q plot for lateral root.

Supplementary Figure 17
**(A)** Phenotypic diversity of population structure and nodal root of 250 accessions. **(B)** Manhattan plot for nodal root **(C)** Q-Q plot for nodal root.

Supplementary Figure 18 Co-expression analysis of genes associated with root traits.
