# Correction: Genetic dissection of root traits in a rice ‘global MAGIC’ population for candidate traits to breed for reduced methane emission

**DOI:** 10.3389/fpls.2025.1692969

**Published:** 2025-09-08

**Authors:** Ripon Kumar Roy, Gopal Misra, Shaina Sharma, Bandana Pahi, Seyed Mahdi Hosseiniyan Khatibi, Kurniawan Rudi Trijatmiko, Sung-Ryul Kim, Jose E. Hernandez, Amelia Henry, Nese Sreenivasulu, Maria Genaleen Q. Diaz, Eureka Teresa M. Ocampo, Pallavi Sinha, Ajay Kohli

**Affiliations:** ^1^ International Rice Research Institute (IRRI), Los Baños, Philippines; ^2^ Bangladesh Rice Research Institute (BRRI), Gazipur, Bangladesh; ^3^ University of the Philippines Los Baños, College, Laguna, Philippines; ^4^ International Rice Research Institute, South Asia Hub, Hyderabad, Telangana, India

**Keywords:** root diameter, root porosity, genome-wide association analysis, superior haplotype, protein-protein interaction, methane emission

Affiliation 1 “International Rice Research Institute (IRRI), Los Baños, Philippines” was erroneously given as “Rice Breeding Innovations, International Rice Research Institute (IRRI), Los Baños, Philippines”. Affiliation 2 “Bangladesh Rice Research Institute (BRRI), Gazipur, Bangladesh” was erroneously given as “Rice Breeding Innovations, Bangladesh Rice Research Institute (BRRI), Gazipur, Bangladesh”. Affiliation 3 “University of the Philippines Los Baños, Laguna, Philippines” was erroneously given as “Institute of Biological Sciences, University of the Philippines Los Baños, Los Baños, Philippines”. Affiliation 4 “Rice Breeding Innovations, International Rice Research Institute, South Asia Hub, Telangana, India” was erroneously given as “International Rice Research Institute, South Asia Hub, Patancheru, Telangana, India”.

Authors “Jose E. Hernandez” and “Eureka Teresa M. Ocampo” were erroneously assigned the affiliation “Institute of Crop Sciences, University of Philippines Los Baños, Los Baños, Philippines”. This affiliation has now been removed.

The author “Sung-Ryul Kim” was erroneously spelled as “Sung Ryul Kim”.

An incorrect **Funding** statement was provided as “The author(s) declare that financial support was received for the research and/or publication of this article. The author acknowledges the financial support of the Indian Council of Agricultural Research (ICAR) under the IRRI-ICAR Work Plan”. The correct **Funding** statement reads:

“The author(s) declare that financial support was received for the research and/or publication of this article. The author acknowledges the financial support of the Indian Council of Agricultural Research (ICAR) under the IRRI-ICAR Work Plan and National Agricultural Technology Program-Phase II Project (NATP-2) BARC, Bangladesh.”

In the **Acknowledgements**, the study incorrectly mentioned support from the Breeding Operations Unit (BOU) and the Enterprise Breeding System (EBS) team at IRRI. The corrected **Acknowledgement** statement reads: “The authors express their sincere gratitude to the IRRI authorities for their valuable support in providing greenhouse facilities for the experiments. We also appreciate the help from the Plant Molecular Biology Laboratory and the Plant Physiology Laboratory at IRRI for their contributions to the experimental work and data processing. We would also like to acknowledge the NATP-2 project for providing a scholarship that supported the author during the course of the experiment.”

In the **Abstract**, the porosity values were mistakenly presented in grams “Porosity (5.344–56.793 g)”. The correct unit of measurement for porosity is percentage “Porosity (5.344–56.793%)”.

In **Results**, first paragraph, the unit of measurement for porosity was incorrectly presented as “grams (g)” instead of “percentage (%)”. The corrected sentence is “In the first experiment, the traits average root porosity (ARP), base root porosity (BRP), and tip root porosity (TRP) ranged from 9.457 to 47.098%, 11.41 to 53.93%, and 5.344 to 43.911%, respectively.”

The same correction has been made in **Results**, second paragraph. The corrected sentence is “In other experiments, ARP, BRP, and TRP ranged from 11.19 to 47.77%, 10.738 to 53.859%, and 7.168 to 56.793%, respectively.”

In **Results**, third paragraph a correction has been made. In the original article, the number of significant MTAs for base root porosity was incorrectly reported as “50” instead of “100”. The corrected sentence is “For Base root porosity, a total of 100 significant MTAs were identified from the GWAS analysis in both experiments.”

The original version of this article has been updated.

