# Supplementary material for: Genetic dissection of root traits in a rice ‘global MAGIC’ population for candidate traits to breed for reduced methane emission
Source: Front Plant Sci. 2025 Jul 9;16:1616424. doi: 10.3389/fpls.2025.1616424 (PMC12283710; doi:10.3389/fpls.2025.1616424)
Supplement: Supplementary Figure 1 — Phenotypic variation and correlation among traits in experiment 1. The correlation plot compares Average Root Porosity (ARP), Base Root Porosity (BRP), Root Diameter (RDia), Tip Root Porosity (TRP), Tiller Number (TN), Root Number (RN), Root Per Tiller (RPT), Shoot Dry Weight (SDW), Root Dry Weight (RDW), Ratio Between Root Shoot (RS ratio), Root Length (RL), Fine Lateral Root (FLR), Thick Lateral Root (TLR), Lateral Root (LR) and Nodal Root (NR). Bar charts show the frequency distributions of each trait on the diagonal. Pearson correlation values are above the diagonal with their corresponding P-values, and scatter plots are below the diagonal. Data correspond to mean values of 250 MAGIC population in two different experiments (a, b). Symbols indicate the significance levels: * **P < 0.01 and ***P < 0.005. [file Image1.pdf]

Genetic dissection of root traits in a rice 'global MAGIC' population as a precursor to breeding for reduced methane emission

Ripon Kumar Roy[1,2,4], Gopal Misra[1], Shaina Sharma[1,4], Bandana Pahi[1,4], Seyed Mahdi Hosseiniyan Khatibi[1], Kurniawan Rudi Trijatmiko[1], Sung Ryul Kim[1], Jose E. Hernandez[5], Amelia Henry[1], Nese Sreenivasulu[1], Maria Genaleen Q. Diaz[3], Eureka Teresa M. Ocampo[5], Pallavi Sinha[1,4], Ajay Kohli[1] *

[1]International Rice Research Institute (IRRI), Los Baños, Philippines

[2]Bangladesh Rice Research Institute (BRRI), Gazipur, Bangladesh

[3]University of Philippines, Los Baños, Philippines

[4]International Rice Research Institute, South Asia Hub, Telangana, India

[5]Institute of Crop Sciences, University of Philippines, Los Baños, Los Baños, Philippines

* **Corresponding author:** ajakoy@yahoo.com

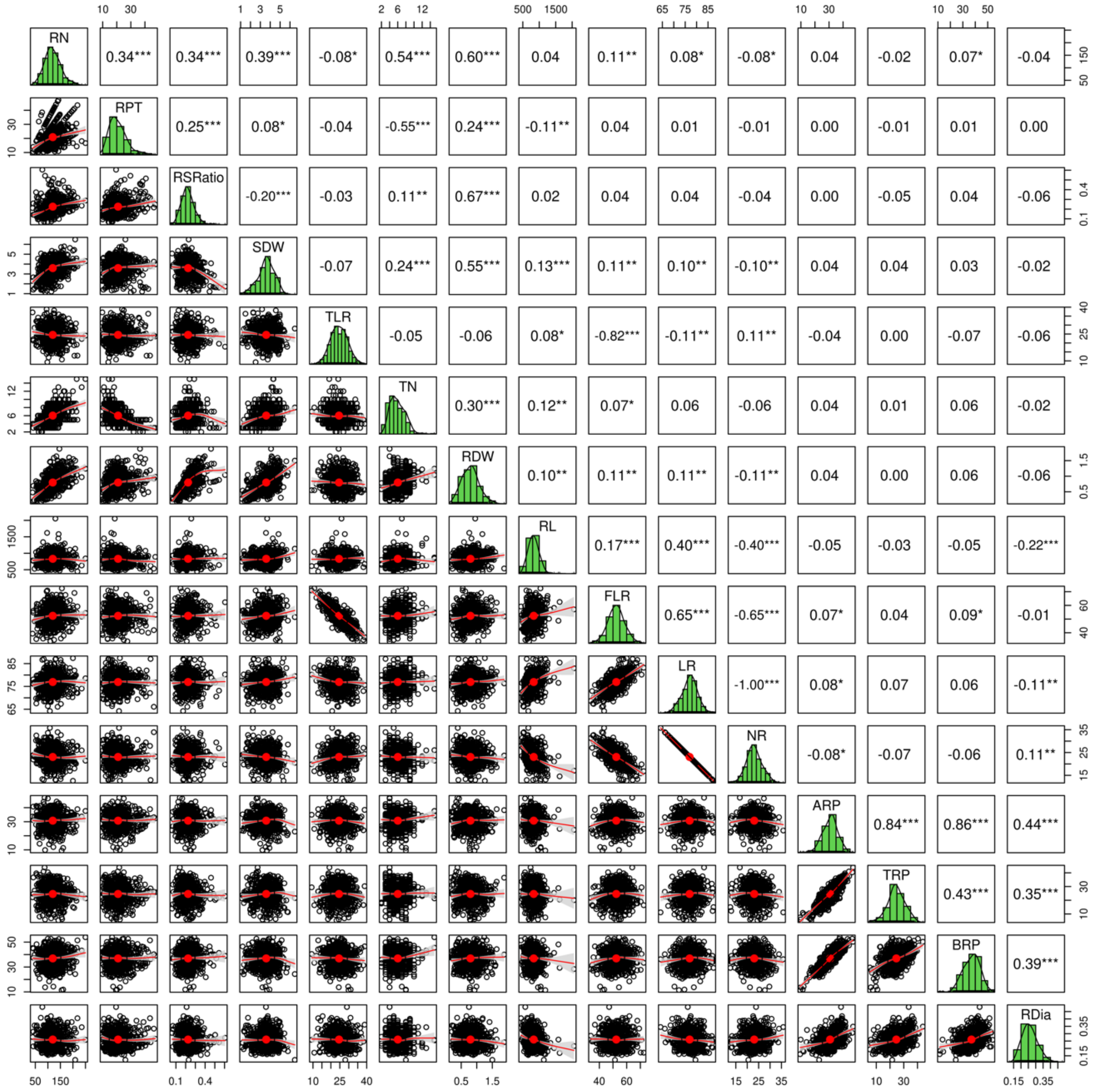

Figure S1. Phenotypic variation and correlation among traits in experiment 1. The corrplot compares Average Root Porosity (ARP), Base Root Porosity (BRP), Root Diameter (Rdia), Tip Root Porosity (TRP), Tiller Number (TN), Root Number (RN), Root Per Tiller (RPT), Shoot Dry Weight (SDW), Root Dry Weight (RDW), Ratio Between Root Shoot (RS ratio), Root Length (RL), Fine Lateral Root (FLR), Thick Lateral Root (TLR), Lateral Root (LR) and Nodal Root (NR). Bar charts show the frequency distributions of each trait on the diagonal. Pearson correlation values are above the diagonal with their corresponding P-values, and scatter plots are below the diagonal. Data correspond to mean values of 250 MAGIC population in two different experiments (a,b). Symbols indicate the significance levels: * **P < 0.01 and ***P < 0.005.

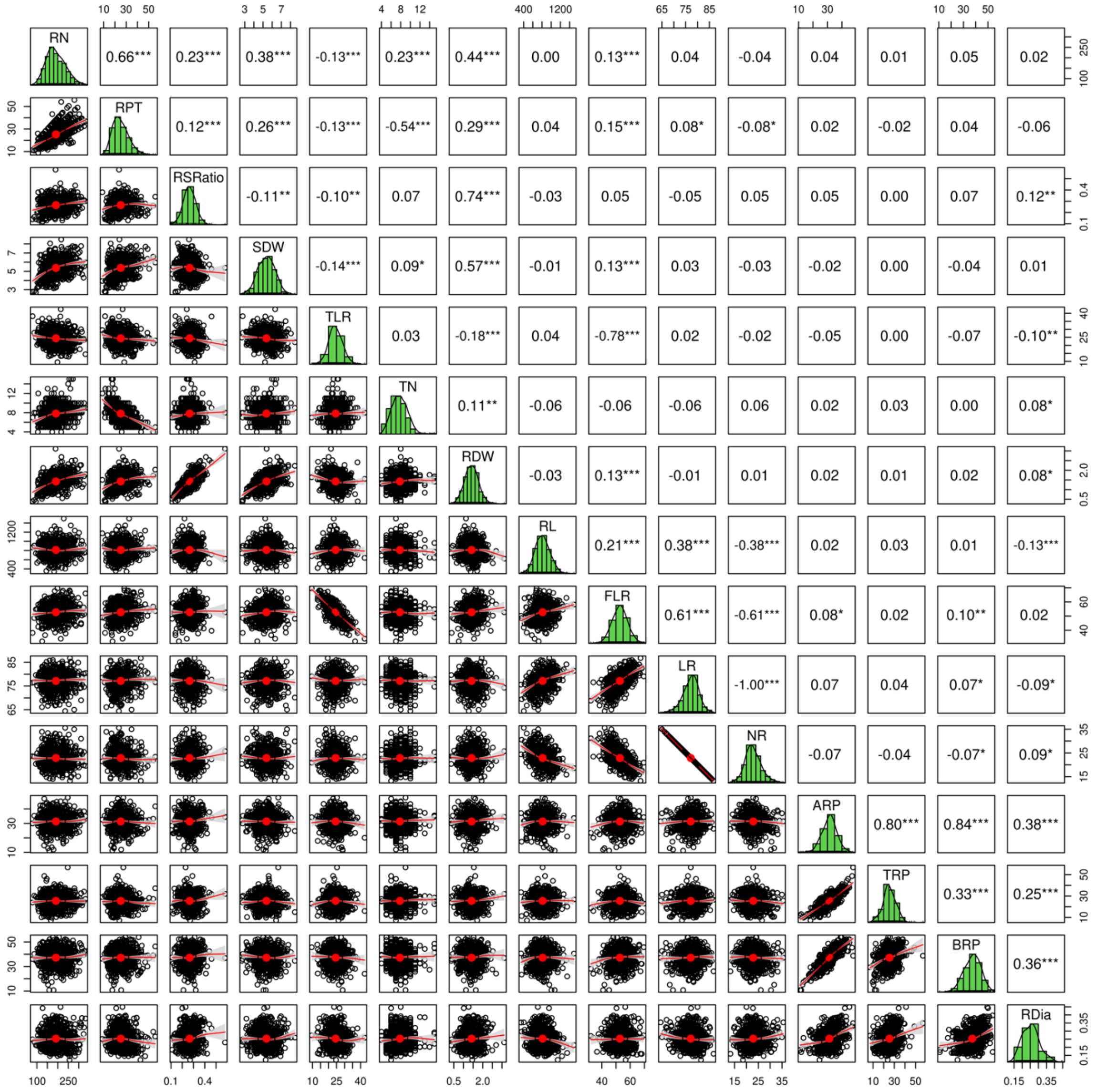

Figure S2. Phenotypic variation and correlation among traits in experiment 2. The corrplot compares Average Root Porosity (ARP), Base Root Porosity (BRP), Root Diameter (Rdia), Tip Root Porosity (TRP), Tiller Number (TN), Root Number (RN), Root Per Tiller (RPT), Shoot Dry Weight (SDW), Root Dry Weight (RDW), Ratio Between Root Shoot (RS ratio), Root Length (RL), Fine Lateral Root (FLR), Thick Lateral Root (TLR), Lateral Root (LR) and Nodal Root (NR). Bar charts show the frequency distributions of each trait on the diagonal. Pearson correlation values are above the diagonal with their corresponding P-values, and scatter plots are below the diagonal. Data correspond to mean values of 250 MAGIC population in two different experiments (a,b). Symbols indicate the significance levels: * **P < 0.01 and ***P < 0.005.

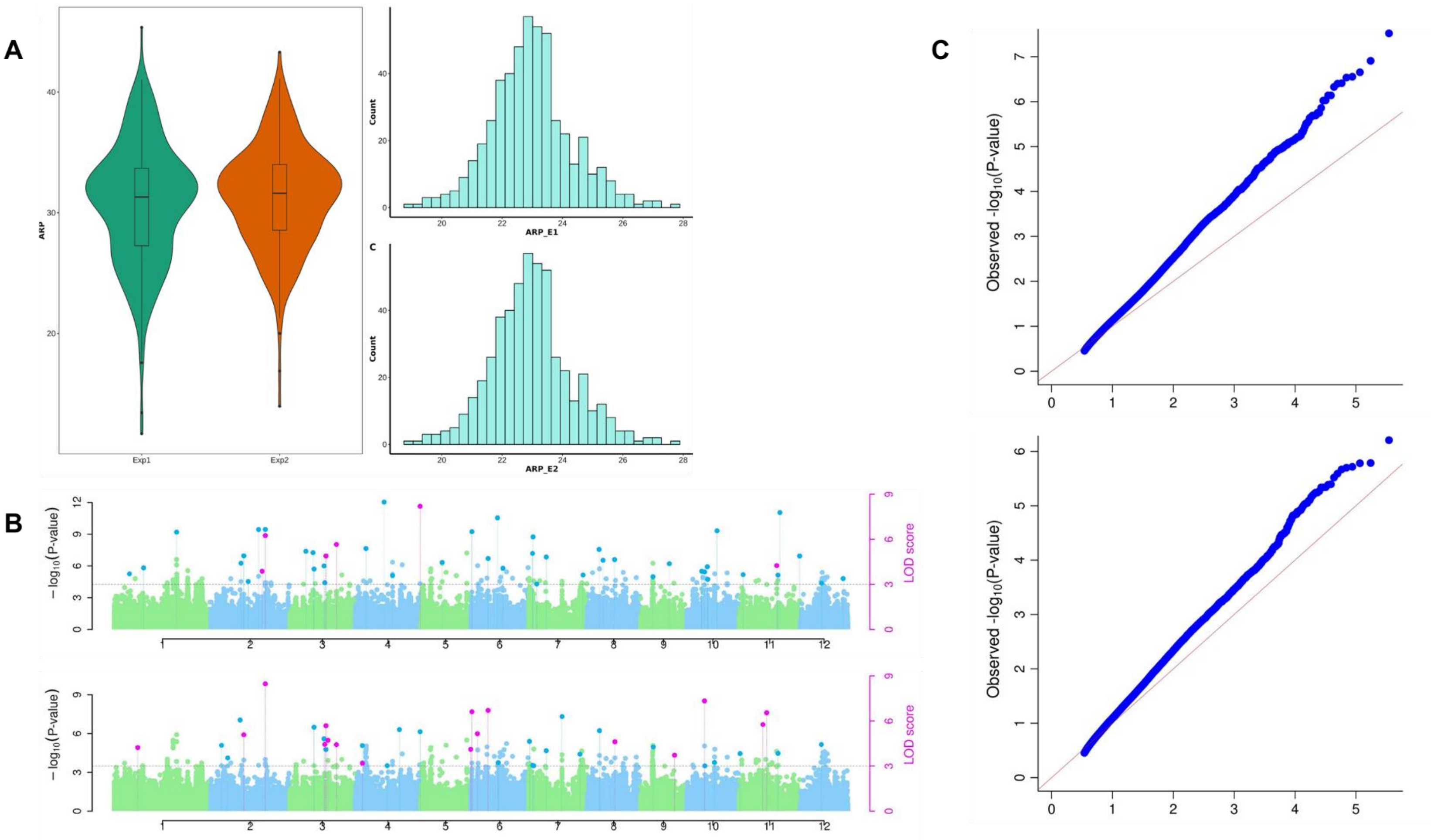

Figure S3. (A) Phenotypic diversity of population structure and average root porosity of 250 accessions. **(B)** Manhattan plot for root length. **(C)** Q-Q plot for average root porosity

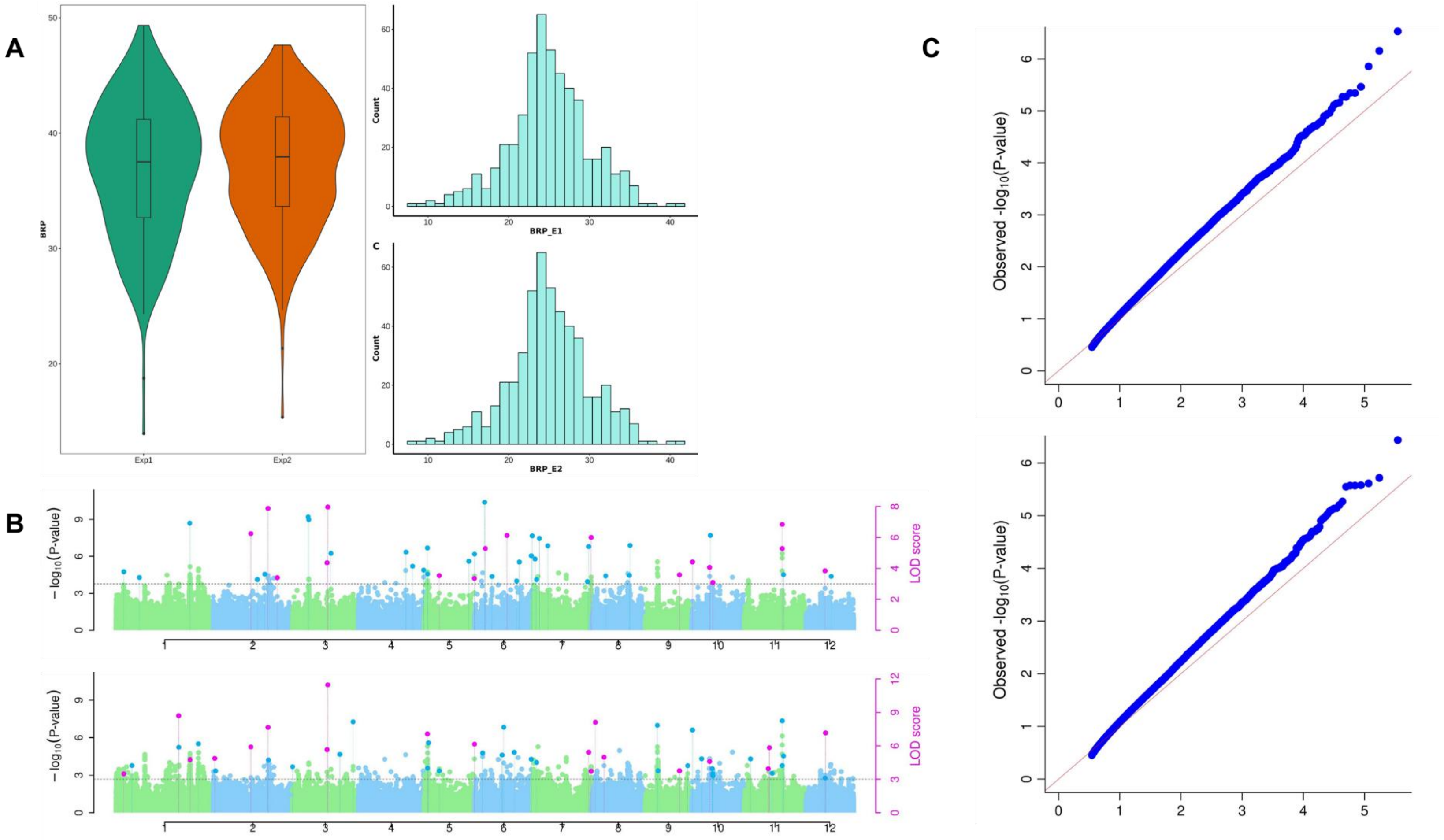

Figure S4. (A) Phenotypic diversity of population structure and base root porosity of 250 accessions. **(B)** Manhattan plot for base root porosity . **(C)** Q-Q plot for base root porosity

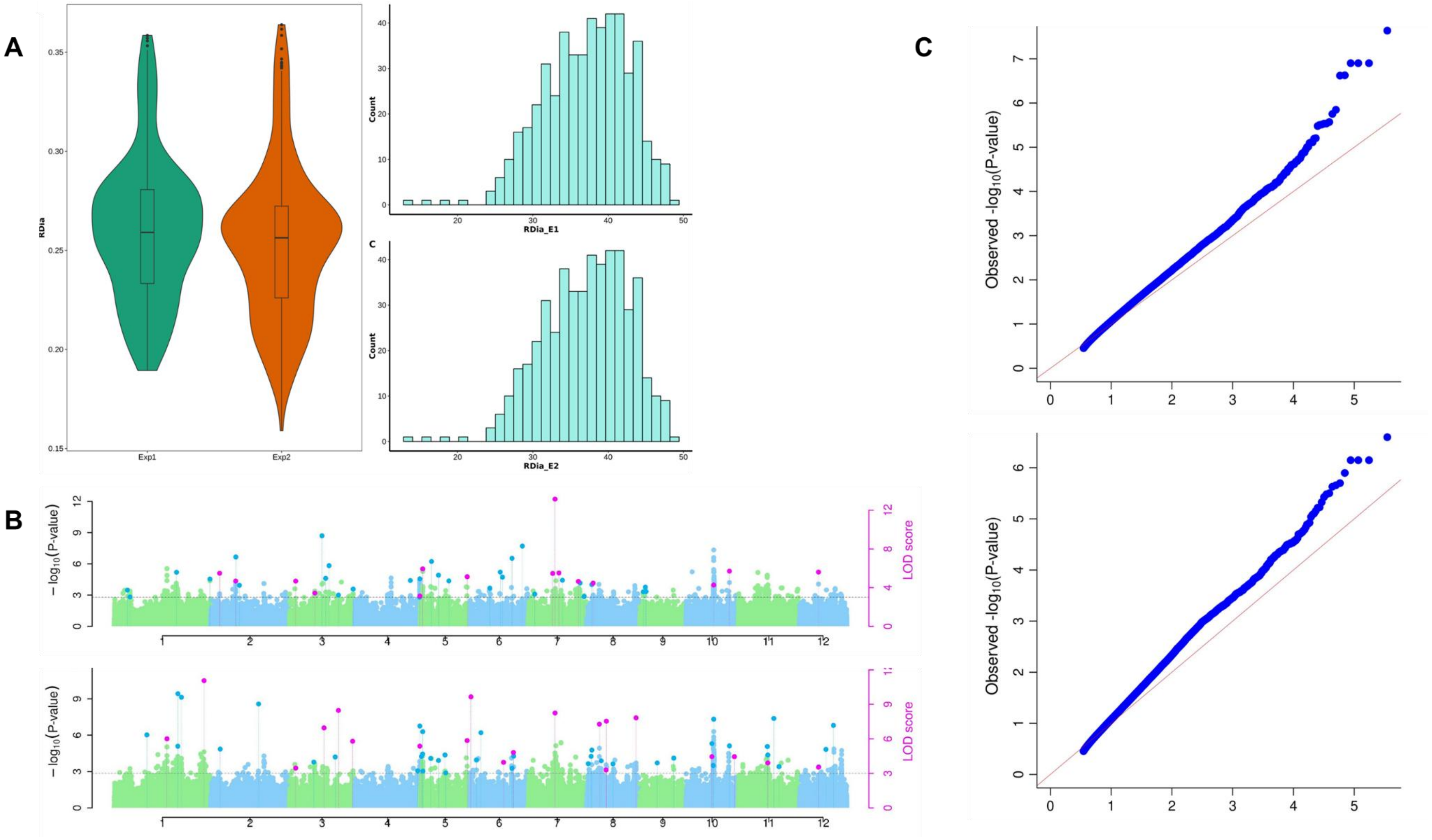

Figure S5. (A) Phenotypic diversity of population structure and root diameter of 250 accessions. **(B)** Manhattan plot for root diameter **(C)** Q-Q plot for root diameter

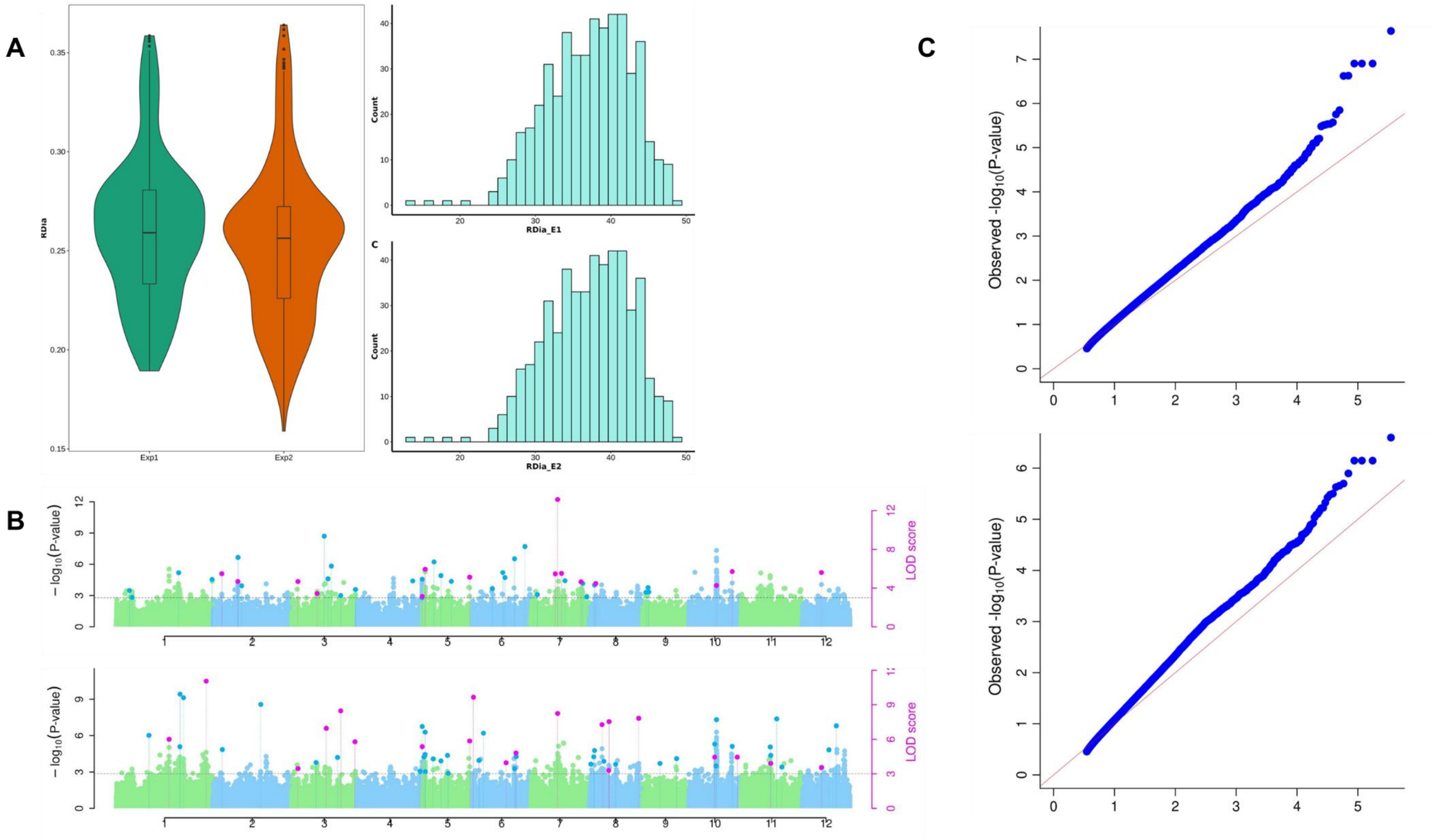

Figure S6. (A) Phenotypic diversity of population structure and tip root porosity of 250 accessions. **(B)** Manhattan plot for tip root porosity **(C)** Q-Q plot for tip root porosity

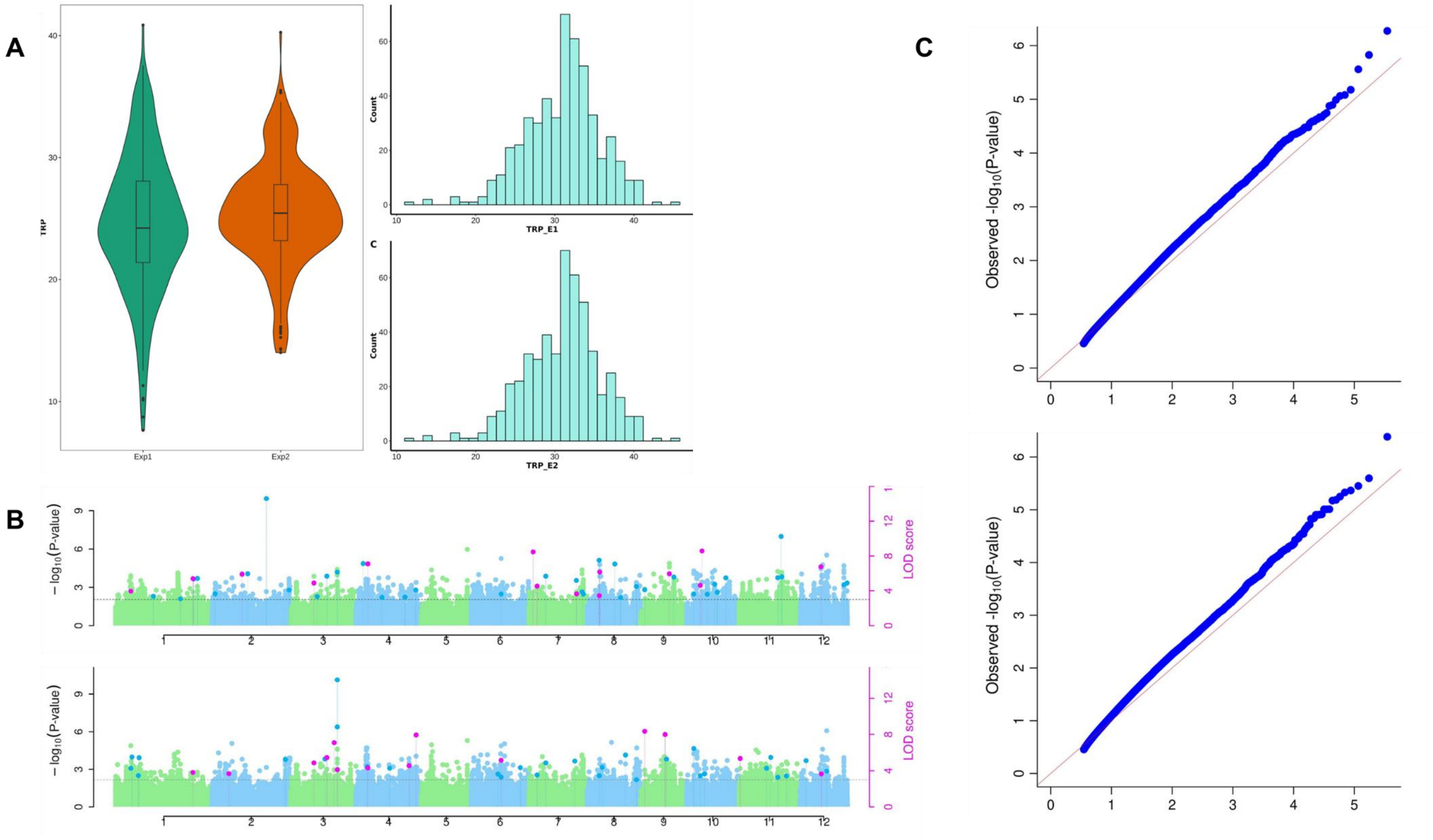

Figure S7. (A) Phenotypic diversity of population structure and tiller number of 250 accessions. **(B)** Manhattan plot for tiller number **(C)** Q-Q plot for tiller number

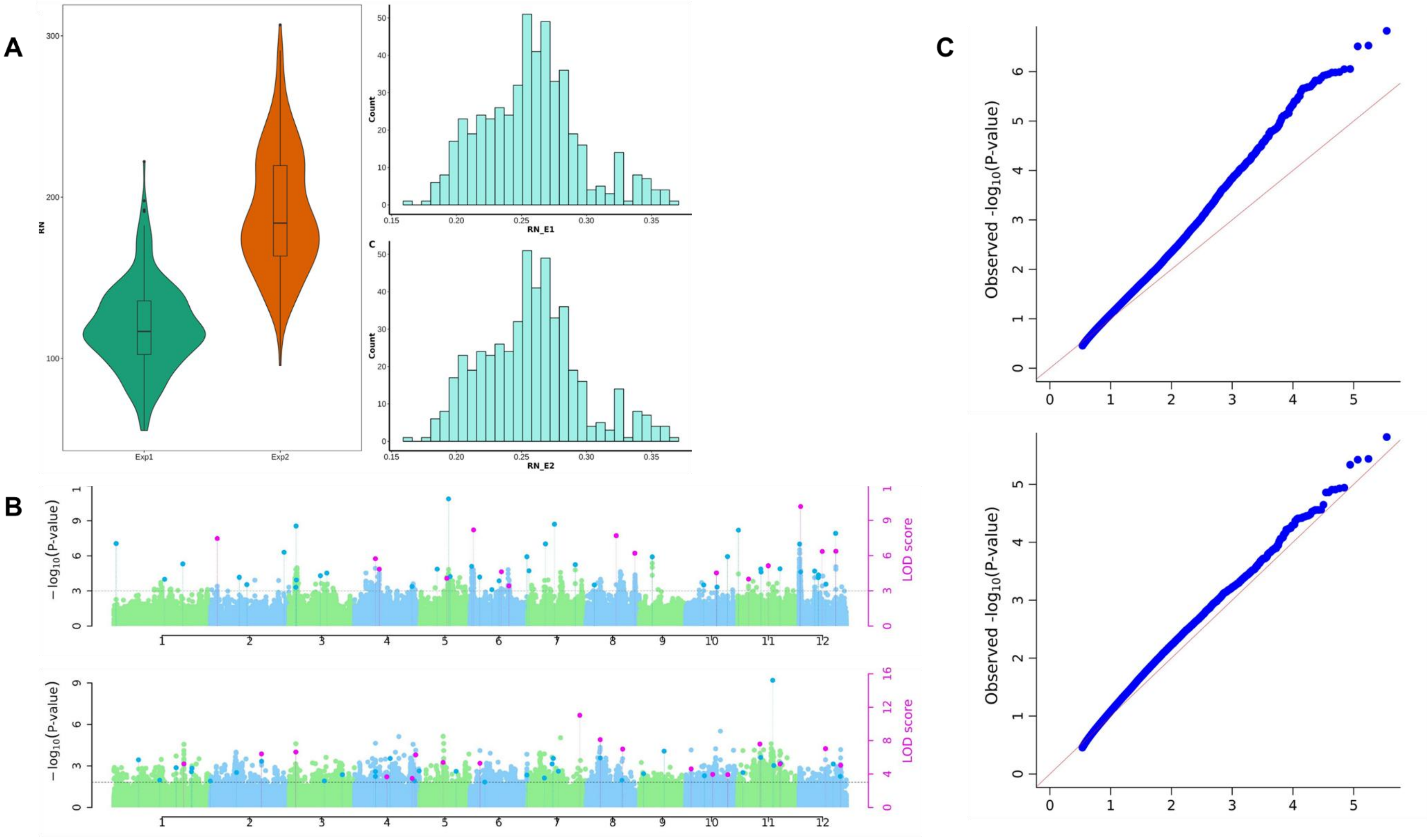

Figure S8. (A) Phenotypic diversity of population structure and root number of 250 accessions. **(B)** Manhattan plot for root number **(C)** Q-Q plot for root number

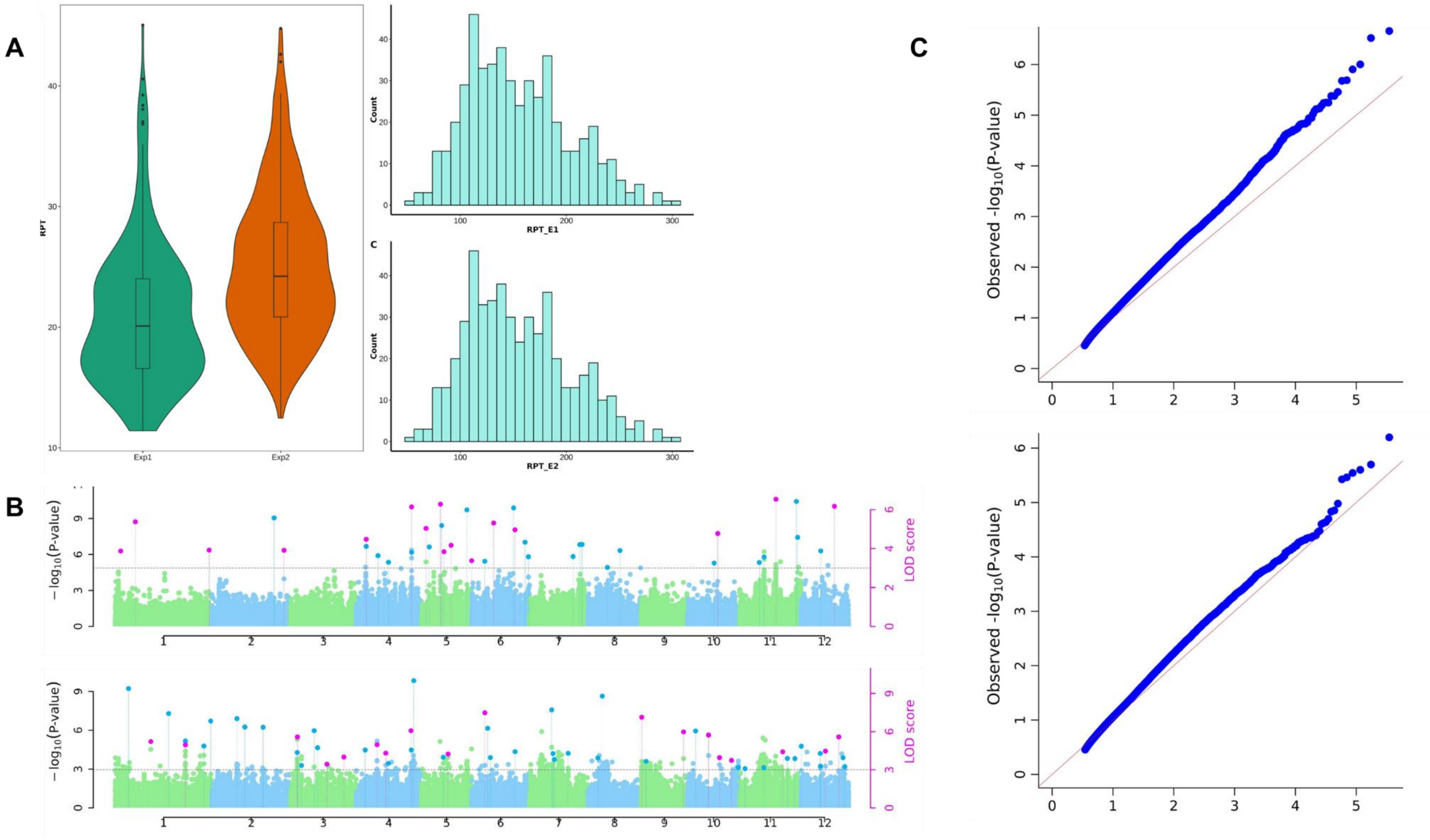

Figure S9. (A) Phenotypic diversity of population structure and root per tiller of 250 accessions. **(B)** Manhattan plot for root per tiller **(C)** Q-Q plot for root per tiller

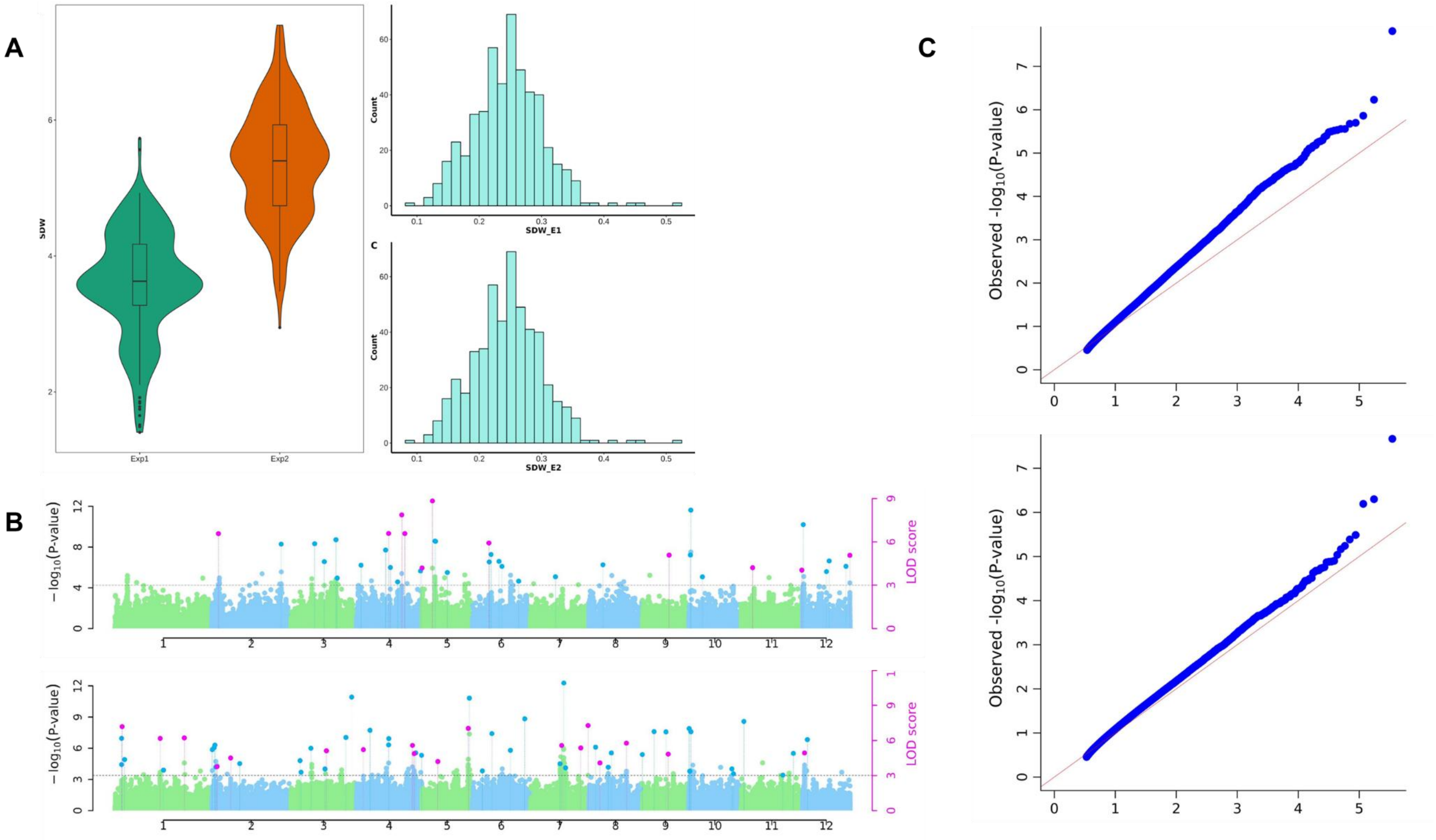

Figure S10. (A) Phenotypic diversity of population structure and shoot dry weight of 250 accessions. **(B)** Manhattan plot for shoot dry weight **(C)** Q-Q plot for shoot dry weight

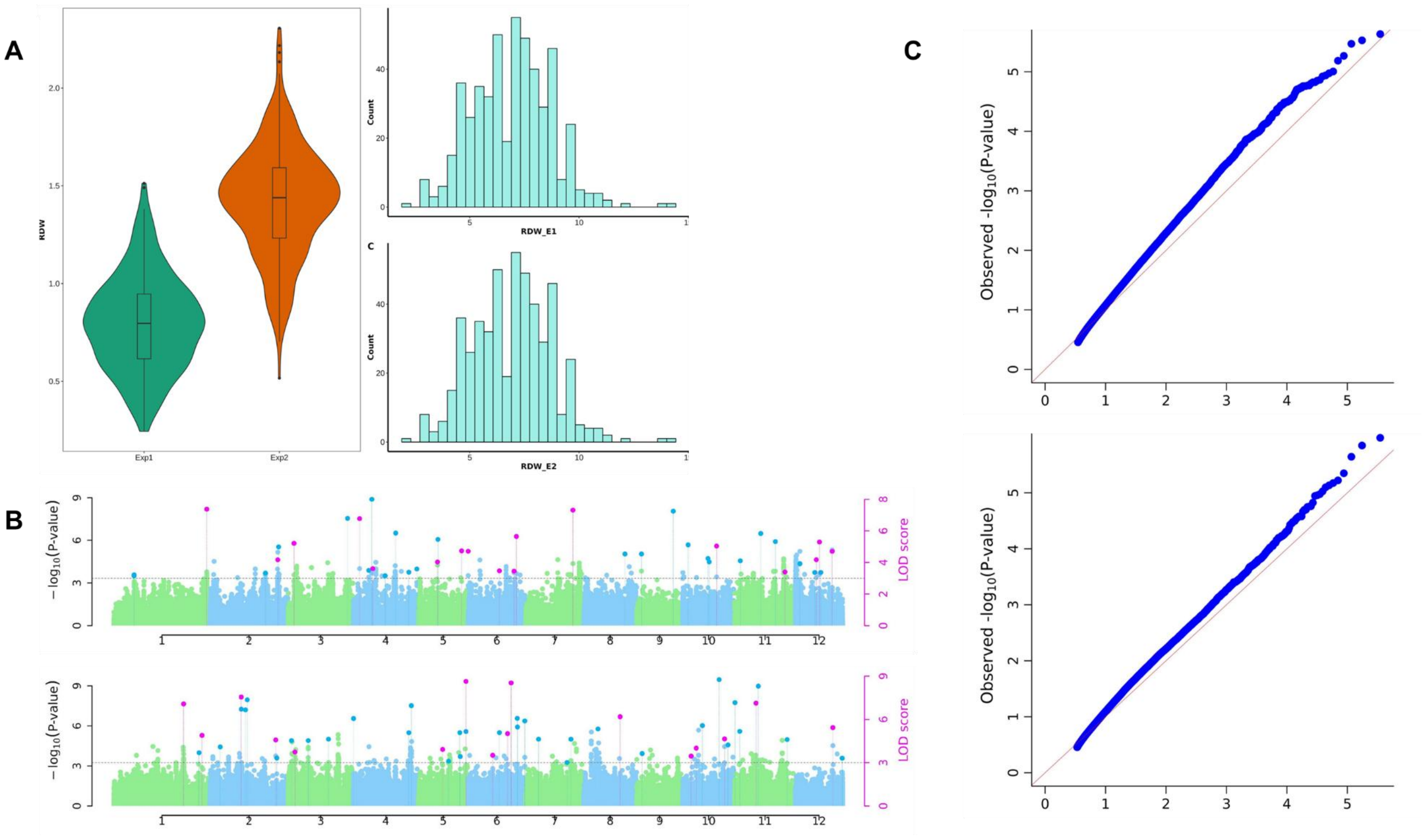

Figure S11. (A) Phenotypic diversity of population structure and root dry weight of 250 accessions. **(B)** Manhattan plot for root dry weight **(C)** Q-Q plot for root dry weight

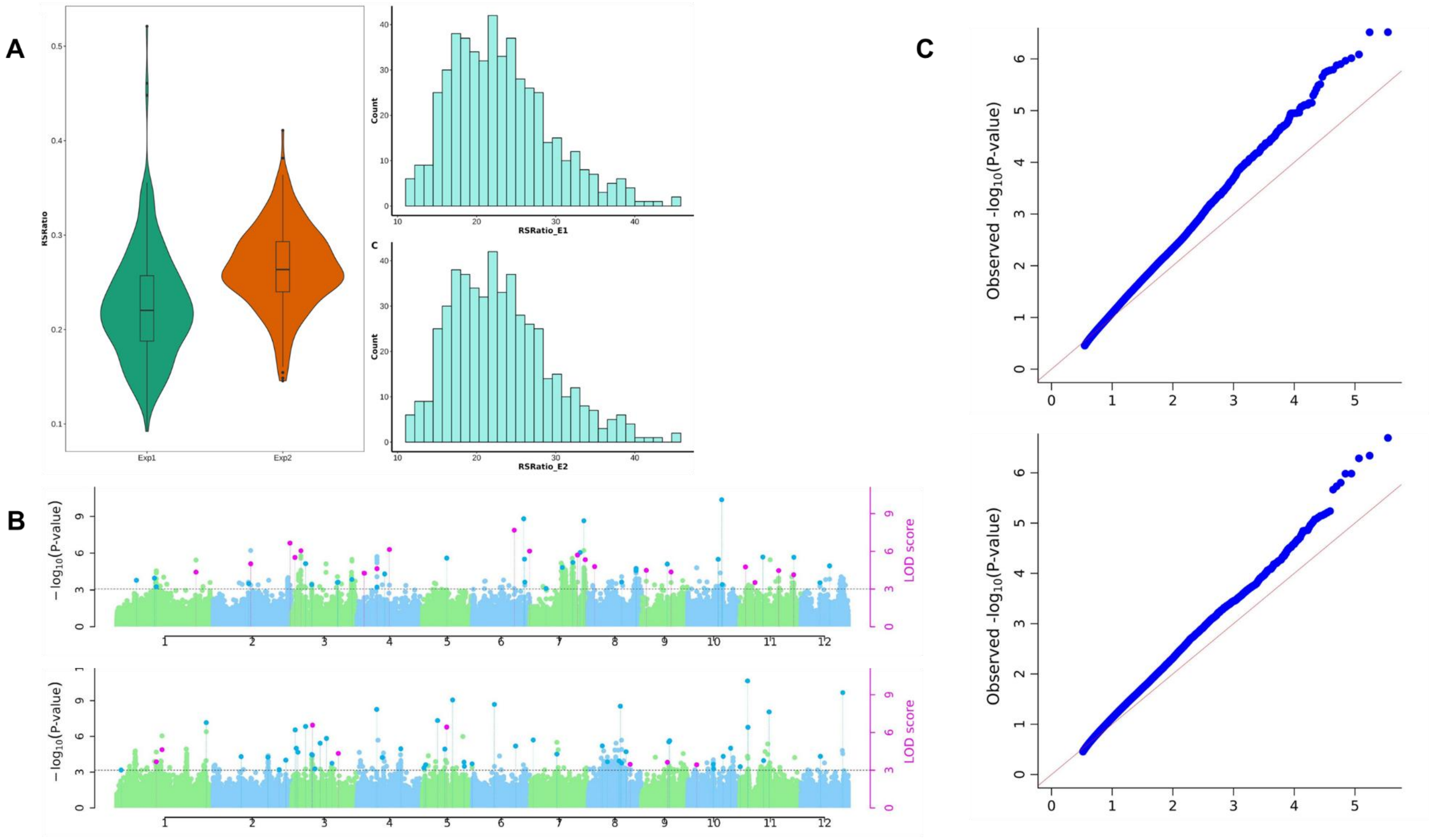

Figure S12. **(A)** Phenotypic diversity of population structure and root shoot ratio of 250 accessions. **(B)** Manhattan plot for root shoot ratio **(C)** Q-Q plot for root shoot ratio

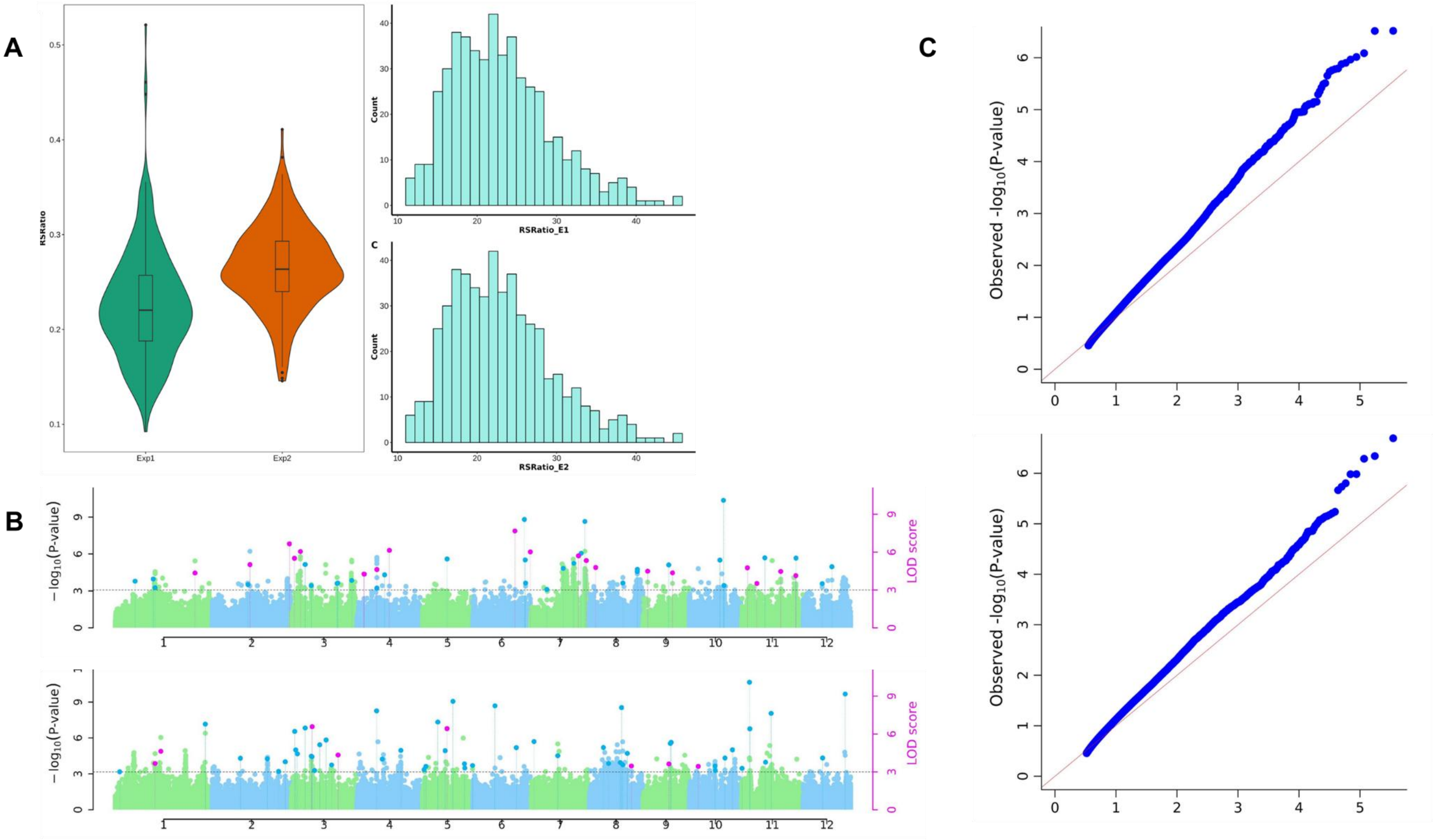

Figure S13. (A) Phenotypic diversity of population structure and root length of 250 accessions. **(B)** Manhattan plot for root length **(C)** Q-Q plot for root length

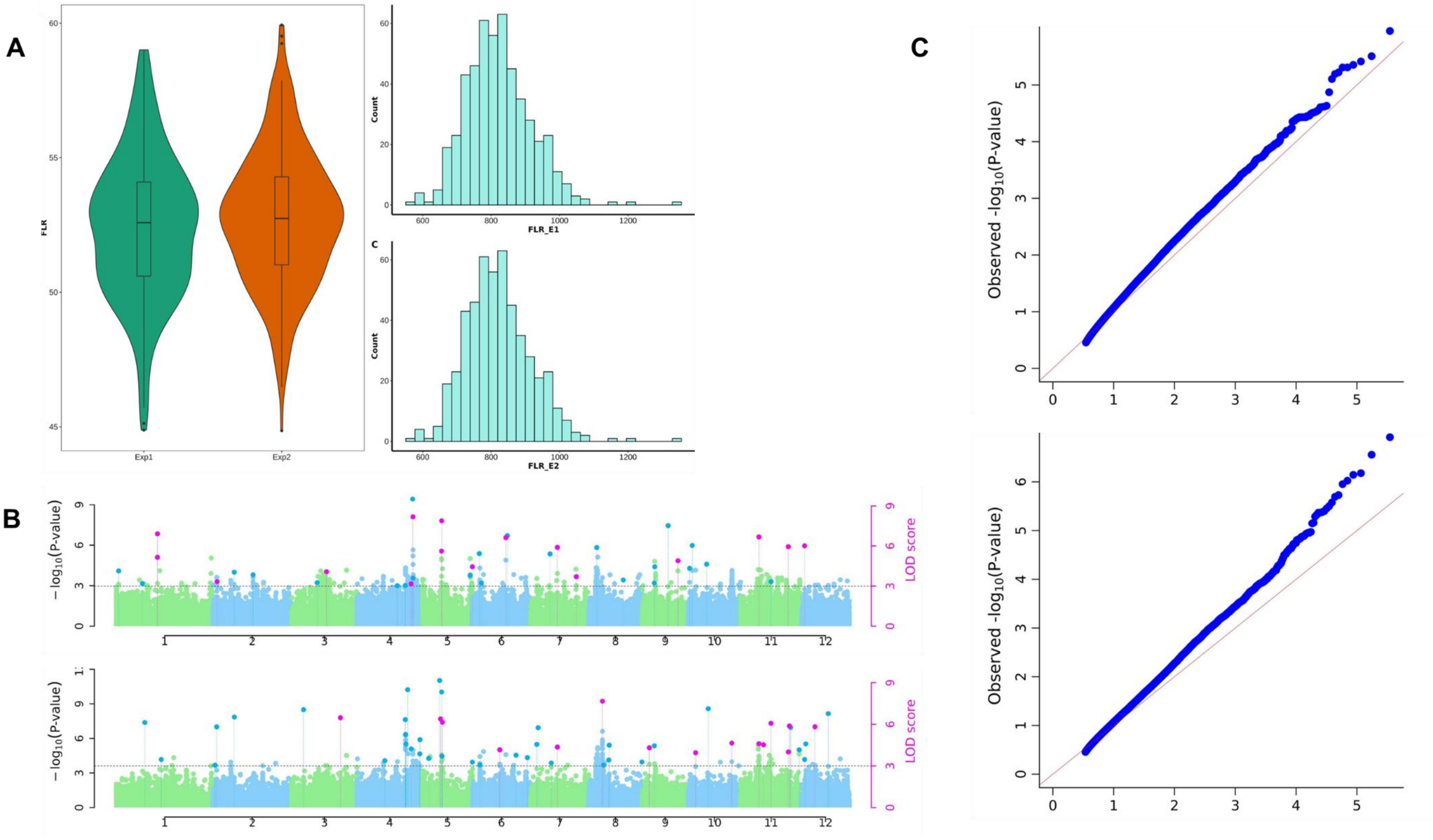

Figure S14. (A) Phenotypic diversity of population structure and fine lateral root of 250 accessions. **(B)** Manhattan plot for fine lateral root **(C)** Q-Q plot for fine lateral root

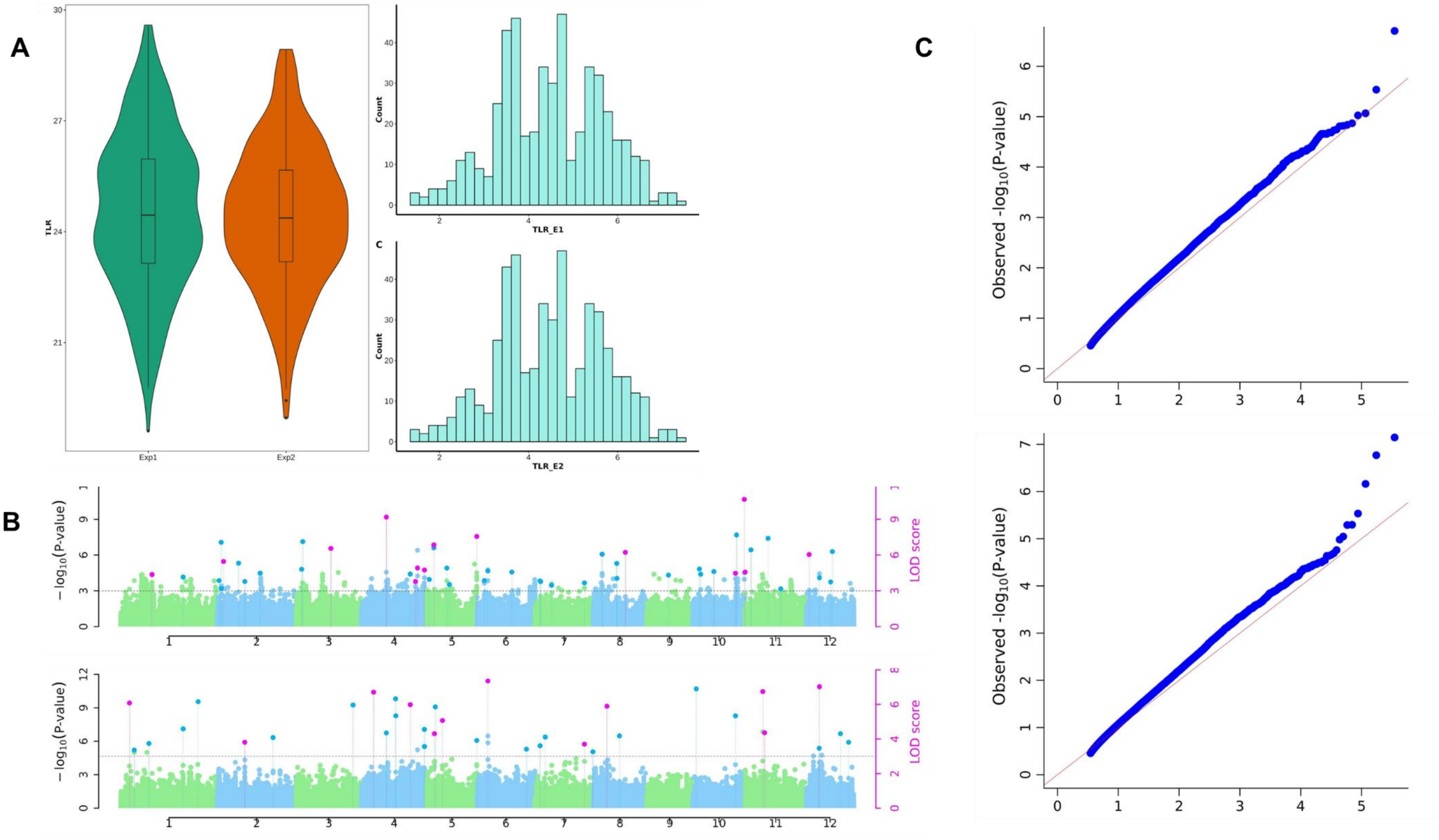

Figure S15. (A) Phenotypic diversity of population structure and thick lateral root of 250 accessions. **(B)** Manhattan plot for thick lateral root (C) Q-Q plot for thick lateral root

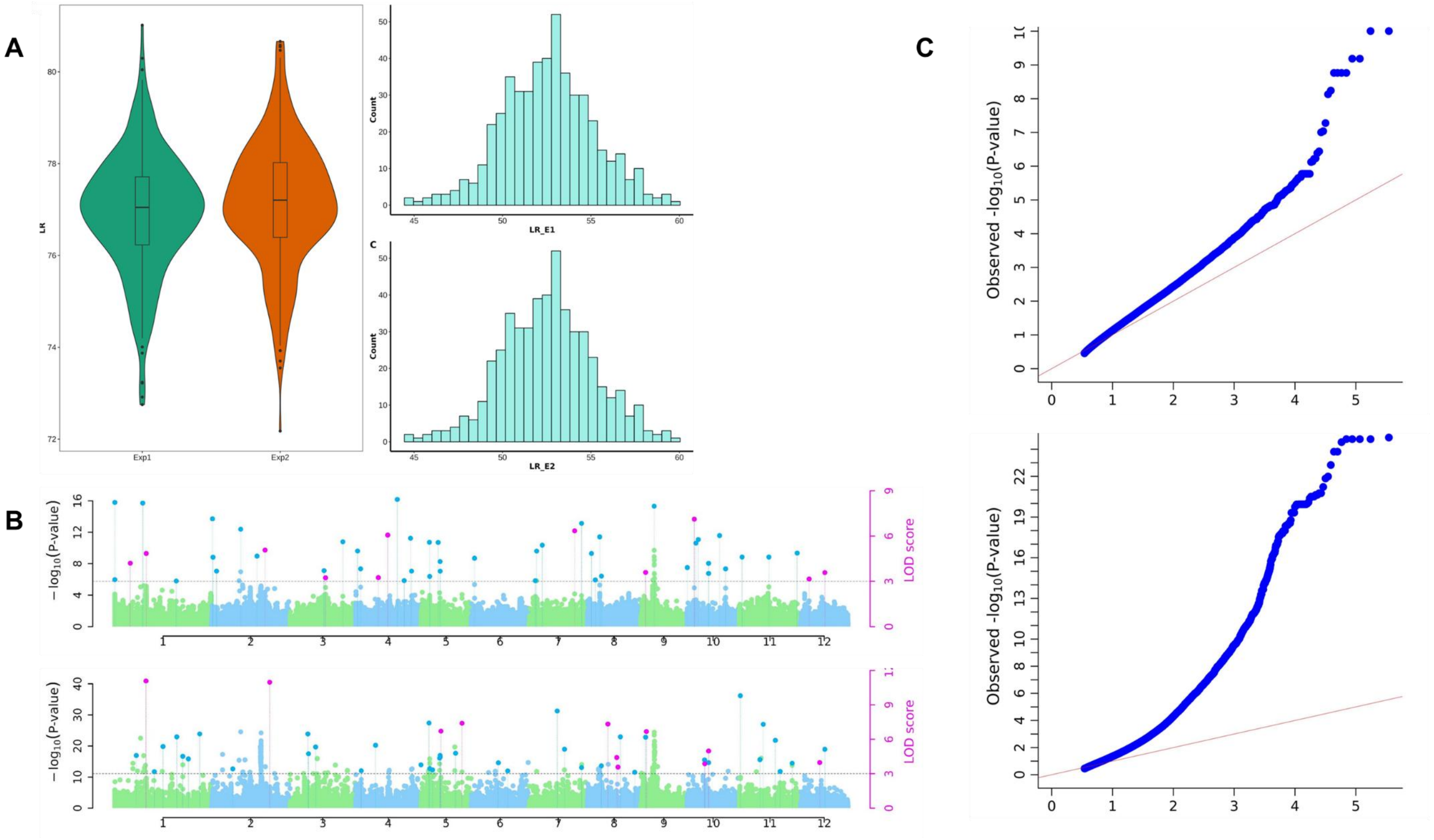

Figure S16. (A) Phenotypic diversity of population structure and lateral root of 250 accessions. **(B)** Manhattan plot for lateral root **(C)** Q-Q plot for lateral root

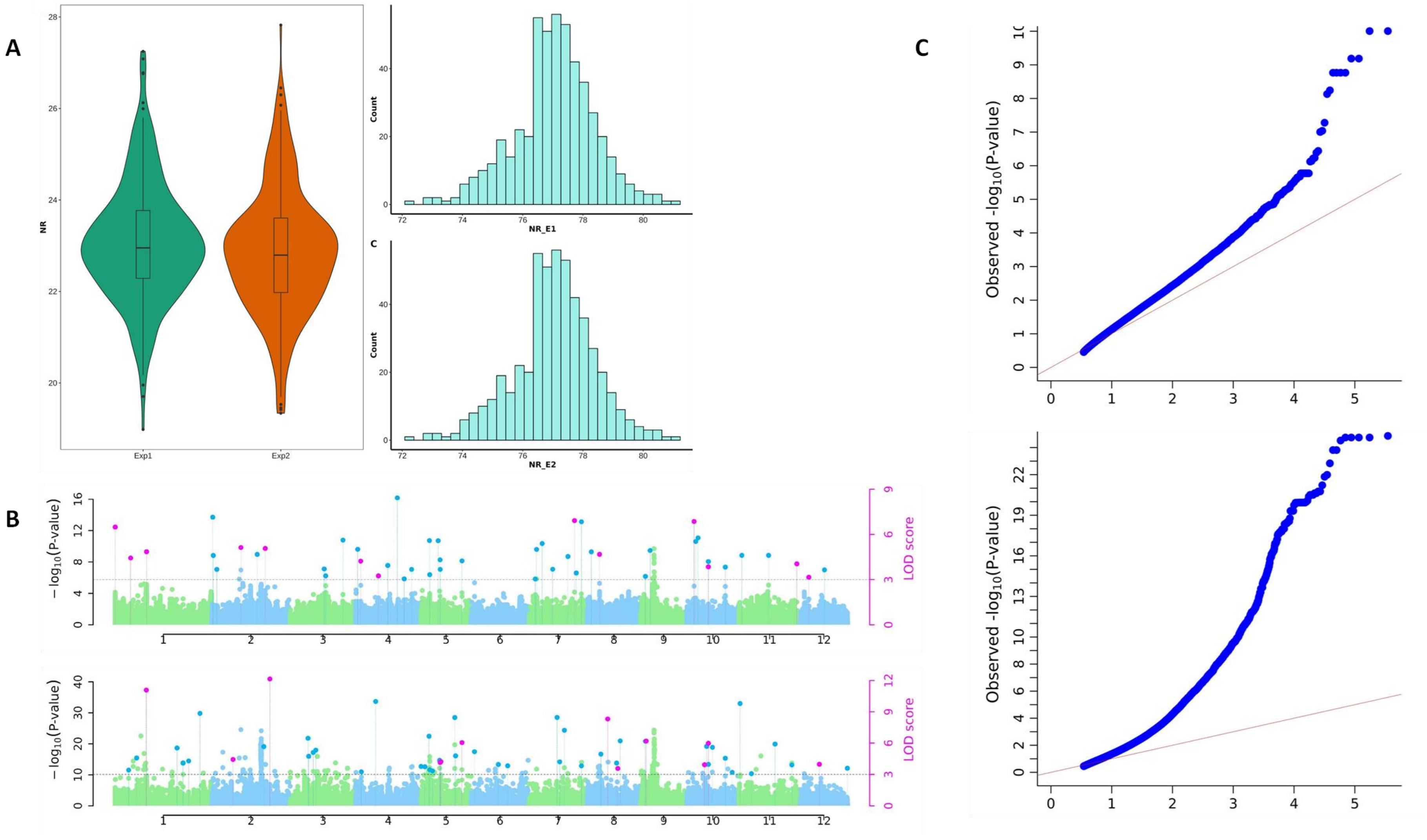

Figure S17. (A) Phenotypic diversity of population structure and nodal root of 250 accessions. **(B)** Manhattan plot for nodal root **(C)** Q-Q plot for nodal root

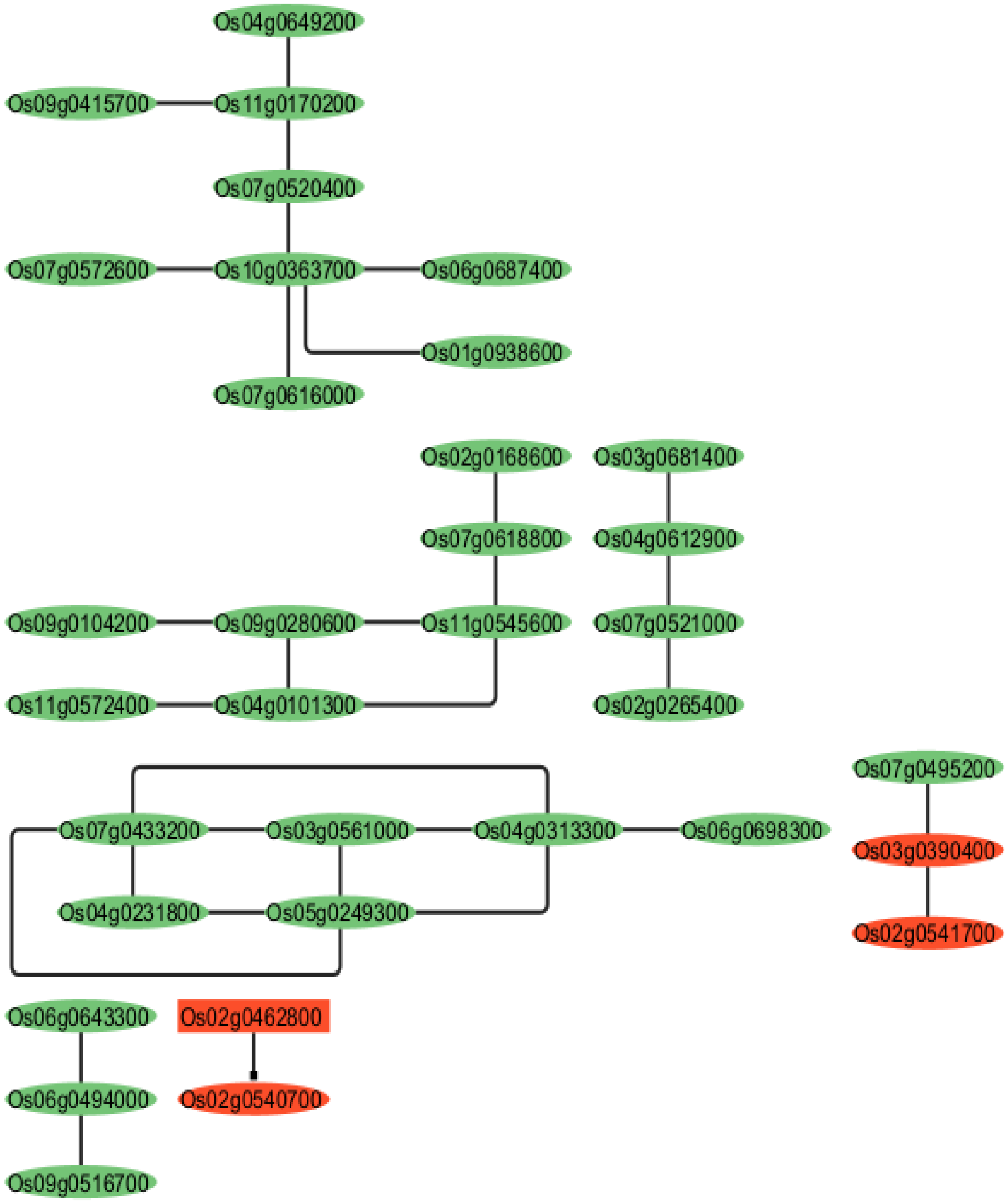

Figure S18. Co-expression analysis of genes associated with root traits
